# Metagenomic Analysis of Hot Springs in Central India Reveals Hydrocarbon Degrading Thermophiles and Pathways Essential for Survival in Extreme Environments

**DOI:** 10.3389/fmicb.2016.02123

**Published:** 2017-01-05

**Authors:** Rituja Saxena, Darshan B. Dhakan, Parul Mittal, Prashant Waiker, Anirban Chowdhury, Arundhuti Ghatak, Vineet K. Sharma

**Affiliations:** ^1^Metagenomics and Systems Biology Laboratory, Department of Biological Sciences, Indian Institute of Science Education and ResearchBhopal, India; ^2^Department of Earth and Environmental Sciences, Indian Institute of Science Education and ResearchBhopal, India

**Keywords:** metagenomics, extremophiles, thermophiles, Indian hot springs, hydrocarbon degradation, Anhoni, Tattapani

## Abstract

Extreme ecosystems such as hot springs are of great interest as a source of novel extremophilic species, enzymes, metabolic functions for survival and biotechnological products. India harbors hundreds of hot springs, the majority of which are not yet explored and require comprehensive studies to unravel their unknown and untapped phylogenetic and functional diversity. The aim of this study was to perform a large-scale metagenomic analysis of three major hot springs located in central India namely, Badi Anhoni, Chhoti Anhoni, and Tattapani at two geographically distinct regions (Anhoni and Tattapani), to uncover the resident microbial community and their metabolic traits. Samples were collected from seven distinct sites of the three hot spring locations with temperature ranging from 43.5 to 98°C. The 16S rRNA gene amplicon sequencing of V3 hypervariable region and shotgun metagenome sequencing uncovered a unique taxonomic and metabolic diversity of the resident thermophilic microbial community in these hot springs. Genes associated with hydrocarbon degradation pathways, such as benzoate, xylene, toluene, and benzene were observed to be abundant in the Anhoni hot springs (43.5–55°C), dominated by *Pseudomonas stutzeri* and *Acidovorax* sp., suggesting the presence of chemoorganotrophic thermophilic community with the ability to utilize complex hydrocarbons as a source of energy. A high abundance of genes belonging to methane metabolism pathway was observed at Chhoti Anhoni hot spring, where methane is reported to constitute >80% of all the emitted gases, which was marked by the high abundance of *Methylococcus capsulatus*. The Tattapani hot spring, with a high-temperature range (61.5–98°C), displayed a lower microbial diversity and was primarily dominated by a nitrate-reducing archaeal species *Pyrobaculum aerophilum*. A higher abundance of cell metabolism pathways essential for the microbial survival in extreme conditions was observed at Tattapani. Taken together, the results of this study reveal a novel consortium of microbes, genes, and pathways associated with the hot spring environment.

## Introduction

Extremophilic microorganisms are known to survive in diverse extreme conditions, such as high or low temperatures, high salinity, acidic and alkaline pH-values, and high radiation (Mirete et al., 2016). Among these extremophilic microbes, thermophiles, and hyper-thermophiles have the ability to survive in environments with very high temperature such as in hot springs, with the help of enzymes that remain catalytically active under such conditions. Detailed genomic analysis of many novel thermophiles isolated from hot springs has revealed their potential applications in industrial and biotechnological processes (Deckert et al., 1998; Sharma et al., 2014; Poli et al., 2015; Saxena et al., 2015; Dhakan et al., 2016). The remarkable genomic versatility and complexity of such largely unculturable extremophilic communities can be accessed using metagenomics and next-generation sequencing technologies (Lopez-Lopez et al., 2013), and has lead to the discovery of many novel thermophilic bacterial and viral high-quality genomes with several prospective applications (Colman et al., 2016; Eloe-Fadrosh et al., 2016; Gudbergsdottir et al., 2016).

A time point metagenomic study carried out for 3 years in three alkaline hot springs of Yellowstone National Park (44–75°C) showed variations in the resident bacterial population at the three sites which had different temperatures. However, no significant changes were observed in microbial diversity in the same samples collected at different time points (Bowen De Leon et al., 2013). The elemental analysis of these hot springs revealed elevated levels of sodium, chloride and fluoride, and absence of iron, cobalt, silver, and other heavy metals. The metagenomic analysis revealed the presence of many photosynthetic bacteria (*Cyanobacteria* and *Chloroflexi*) and methanogenic archaea (*Methanomassiliicoccus* and *Methanocella)* in the hot spring samples. In another study based on 16S rRNA amplicon sequencing of metagenomic samples from Sungai Klah (SK) hot spring in Malaysia (50–110°C, pH 7–9), Firmicutes and Proteobacteria were found as the most abundant phyla (Chan et al., 2015). Elements like aluminum, arsenic, boron, chloride, fluoride, iron, and magnesium were found at higher levels in the hot spring sample, whereas other heavy metals such as lead, mercury, chromium, copper, etc., were below the limits of quantification. The presence of genes for sulfur, carbon, and nitrogen metabolism suggested metabolic and functional diversity among the microbiome species. The enrichment of carbon metabolism pathway in the SK hot spring was attributed to the high total organic content due to plant litter observed at this site. The major taxa found to be dominant in other alkaline hot springs globally were *Fischerella, Leptolyngbya, Geitlerinema* (Coman et al., 2013), *Stenotrophomonas*, and *Aquaspirillum* (Tekere et al., 2011). However, no significant correlation has been observed between the microbial diversity and geochemical characteristics of the hot springs in the above-mentioned studies.

Metagenomic analysis of an acidic hot spring, *El coquito* in the Colombian Andes, reported the presence of transposase-like sequences involved in horizontal gene transfer and genes for DNA repair system (Jimenez et al., 2012). The hot springs showed a higher proportion of Gammaproteobacteria and Alphaproteobacteria. In other reports focusing on acidic hot springs, the major taxa found to be dominant were *Verrucomicrobia* and *Acidithiobacillus* spp. (Mardanov et al., 2011; Menzel et al., 2015). Many studies have also revealed the presence of genes encoding for enzymes of biotechnological interest, such as hydrolases, xylanases, proteases, galactosidases, and lipases (Ferrandi et al., 2015; Littlechild, 2015). Some studies focusing on the metagenomic analysis of hot springs located in India have reported *Bacillus licheniformis* (Mangrola et al., 2015), *Bacillus megaterium, Bacillus sporothermodurans, Hydrogenobacter* sp., *Thermus thermophilus, Thermus brockianus* (Bhatia et al., 2015), *Clostridium bifermentans, Clostridium lituseburense* (Ghelani et al., 2015), *Opitutus terrae, Rhodococcus erythropolis*, and *Cellovibrio mixtus* (Mehetre et al., 2016) as major bacterial genera. Genes for stress responses and metabolism of aromatic and other organic compounds have been identified by preliminary functional analysis of these sites (Mangrola et al., 2015; Mehetre et al., 2016).

The Geological Survey of India has identified about 340 hot springs located in different parts of India, which are characterized by their orogenic activities (Chandrasekharam, 2005; Bisht et al., 2011). All these hot springs have been classified and grouped into six geothermal provinces on the basis of their geo-tectonic setup. Geothermal resources along Son-Narmada lineament viz. Anhoni-Samoni and Tattapani form the most promising resource base in central India (Shanker, 1986). This is one of the most important lineaments/rifted structure of the sub-continent. It runs across the country in an almost East-West direction and has a long history of tectonic reactivation (Pal and Bhimasankaram, 1976). It contains several known thermal spring areas, the most interesting one being those situated at Tattapani and Anhoni (Bisht et al., 2011). Given the large size and geographical diversity of India, the metagenomic studies from Indian hot springs are still in the infancy stage and more detailed and comprehensive studies are essential to unravel the unknown and untapped microbial and functional diversity of this region. The aim of the study was to perform a comprehensive metagenomic analysis of samples collected from seven different sites at three hot spring locations (Badi Anhoni, Chhoti Anhoni, and Tattapani) situated in two distinct geographical regions, Anhoni and Tattapani, in central India. The hot springs in this study have a temperature range from 43.5 to 98°C and neutral to slightly alkaline pH-values (7.0–7.8). Although, hot springs with similar temperature and pH-values have been studied globally for their phylogenetic and functional characteristics, the geographical location of the currently studied hot springs makes them unique in their geochemical setup. 16S rRNA V3 hypervariable region amplicon sequencing and shotgun metagenome sequencing of all the samples was carried out using Illumina NextSeq 500 for the exploration of microbial communities in the sample and gaining new insights into genes, enzymes, and metabolic pathways contributing to their survival in the thermophilic environment.

## Materials and methods

### Site description and sample collection

The hot springs namely Badi Anhoni and Chhoti Anhoni are located (22.65° N latitude and 78.36° E longitude) ~2 km apart from each other, near the hill station of Pachmarhi in the state of Madhya Pradesh, India (Supplementary Figure 1). Anhoni hot springs are aligned along a prominent geological fracture zone running through that region. A surface hot spring site is located in the Chhoti Anhoni region. In addition, a few boreholes to a depth of ~635 m have been drilled here as a part of petroleum oil exploration and to study the temperature regime and rock segments by the Geological Survey of India, in which presence of inflammable gases (~80% methane) has also been observed (Pandey and Negi, 1995; Sarolkar, 2010; Vaidya et al., 2015). The presence of interlayer basic silts and volcanic tuffs underlain by basic intrusive rock are also reported under these boreholes (Pandey and Negi, 1995). Water samples were collected from two sites at Chhoti Anhoni region, (i) sample from a depth of ~1 m from the surface of the hot spring (referred to as “CAN”) which had a temperature of 43.5°C, and (ii) sample from the free flowing water of the outlet of a borehole (temperature 52.1°C) which was among the several boreholes drilled (reported to be up to 635 m in depth) at Chhoti Anhoni site and was labeled as CAP (Chhoti Anhoni Petroleum). The third sample was collected at a depth of ~1 m from the surface of Badi Anhoni (BAN) sites.

The Tattapani hot spring field is ~700 km away from the Anhoni hot springs and is located in the Sarguja district of Chhattisgarh state, India (23.41°N latitude and 83.39°E longitude). The temperature of different reservoirs at this site has been reported to be as high as 230 ± 40°C at a depth of 2 km and 112 ± 30°C at a depth of 1 km (Vaidya et al., 2015). Among the 26 boreholes that had been drilled here by the Geological survey of India, the ones with distinctively high temperatures (61.5–98°C, up to 325 m deep) were chosen for sample collection (Sarolkar and Das, 2006). Multiple water samples were collected from different physical locations (TAT-1, TAT-2, TAT-3, and TAT-4) and depths, and were pooled to make four distinct samples from each site (~5 L each).

Thus, including three samples from Anhoni and four samples from Tattapani, a total of seven samples were collected. All the water samples were collected in separate sterile plastic carboys (2 L volume) which were rinsed with 0.05% bleach solution for disinfection. A total of 5 L water sample was collected from each site and brought to the laboratory within 12–18 h of collection at 4°C. The samples were stored at −20°C and processed for the extraction of metagenomic DNA within a week. The sample description and physicochemical properties recorded on-site are summarized in Table 1.

**Table 1:** **Physico-chemical properties of the hot spring samples recorded on-site**.

	**Sample name**	**Temperature (°C)**	**pH**	**Total dissolved solids (ppm)**
Anhoni	BAN	55	7.8	690
	CAN	43.5	7.5	590
	CAP	52.1	7.8	620
	Mean	50.2 ± 5.98	7.475 ± 0.34	720 ± 116.62
Tattapani	TAT-1	98	7.5	880
	TAT-2	61.5	7.6	600
	TAT-3	69	7	700
	TAT-4	67	7.8	700
	Mean	73.88 ± 16.39	7.7 ± 0.17	633.3 ± 51.31
Mann-Whitney	Tattapani vs.	0.057	0.571	0.171
*U*-test	Anhoni			

*Six samples (BAN, CAN, TAT-1, TAT-2, TAT-3, and TAT-4) were collected from a depth of about one meter from the surface and one sample (CAP) was collected from the outlet of a ~635 m deep borehole*.

The seven samples considered in this study were divided into two groups, “Anhoni” and “Tattapani,” based on their geographical location for analysis. Hence, the samples collected from Chhoti Anhoni (CAP and CAN) and Badi Anhoni (BAN) sites are referred to as “Anhoni” and the samples collected from the Tattapani hot spring location (TAT-1, TAT-2, TAT-3, and TAT-4) are collectively referred to as “Tattapani” in this study.

### Elemental analysis of the sampling sites

Approximately 250 ml of each water sample was preserved on-site by mixing with 1:100 v/v 5% HNO_3_ for elemental analysis. Dissolved major and trace elements were analyzed using an Inductively Coupled Plasma Mass Spectrometer (ICPMS, iCAPQ—Thermo Fisher Scientific, USA) in accordance with the United States Geological Survey protocol (Hannigan and Basu, 1998; Hannigan et al., 2001) at the Indian Institute of Science Education and Research (IISER) Bhopal, India in a class 10,000 clean lab with class 1000 clear zones. The following elements were analyzed in each sample—Li, Be, B, Mg, Al, Si, K, Ca, V, Cr, Mn, Fe, Co, Ni, Cu, Zn, Se, Sr, Mo, Cd, Cs, Ba, La, Ce, Pb, Hg, and S. All samples were spiked with an internal standard of 10 ppb In, Re, and Bi for internal calibration and the final solution was an undiluted solution in 2% supra pure grade HNO_3_. Ten, hundred, and thousand ppb solutions of the sample elements were prepared and standardized against high-grade multi-elemental standards. These solutions were then used as standards for measurements of the seven water samples of this study. Helium was used as a collision gas to reduce interference of argon oxide ions. Suprapure HCl (5%) was used as a backwash during analyses of Hg and S, while 5% supra pure HNO_3_ was used as a backwash for all other elements. Both Hg and S were measured as separate individual experiments and blanks were measured in between each sample for these two elements to ensure zero memory from previous samples. Analytical uncertainties are <5% for all elements analyzed for this study.

### Metagenomic DNA extraction

In the lab, each sample was filtered through a 1.2 μm pore size membrane filter to remove any traces of debris and coarse particles from the collected water. The filtrate was then passed through a 0.2 μm pore size membrane filter to entrap the prokaryotic cells on the filter. The filter membrane was cut into pieces and subjected to metagenomic DNA extraction using Metagenomic DNA isolation kit for water (Epicentre, Wisconsin, USA) according to the manufacturer's protocol with some modifications to increase the yield and purity of the extracted DNA sample. The modifications included the addition of 100 μl 5M NaCl to 700 μl of isopropanol for efficient precipitation of DNA. The DNA pellet was resuspended in 50 μl of 10 mM Tris (pH 8.5) and evaluated on Genova nanodrop micro-spectrophotometer (Jenway, Bibby Scientific Limited, UK) for purity and Qubit 2.0 fluorometer using Qubit HS dsDNA kit (Life technologies, USA) for quantification.

### 16S rRNA and shotgun metagenome sequencing

The purified DNA samples were used as a template for generating the 16S rDNA V3 amplicon library. The primers used for amplification were 5′TCGTCGGCAGCGTCAGATGTGTATAAGAGACAGCCTACGGGAGGCAGCAG3′ (341F-ADA) and 5′GTCTCGTGGGCTCGGAGATGTGTATAAGAGACAGATTACCGCGGCTGCTGGC3′ (534R-ADA). The underlined regions in the above sequences are the Illumina Nextera XT adapter overhangs, whereas the non-underlined regions are the primer sequences known to target eubacterial 16S rRNA V3 region (Wang and Qian, 2009; Soergel et al., 2012). The optimized PCR conditions were: initial denaturation at 94°C for 5 min, followed by 35 cycles of denaturation at 94°C for 30 s, annealing at 69°C for 30 s, extension at 72°C for 30 s, and a final extension cycle at 72°C for 5 min. Recombinant *Taq* DNA polymerase (Life technologies, USA) was used and 5% DMSO was added to the master mix to enhance the concentration of amplified product from the GC-rich metagenomic template. The amplified products were evaluated on 2% w/v agarose gel, purified using Agencourt Ampure XP kit (Beckman Coulter, USA) and amplicon libraries were prepared by following the Illumina 16S metagenomic library preparation guide. The shotgun metagenomic libraries were prepared using Illumina Nextera XT sample preparation kit (Illumina Inc., USA) using the manufacturer's protocol. Both the libraries were evaluated on 2100 Bioanalyzer using Bioanalyzer DNA 1000 kit for amplicon and High Sensitivity DNA kit for metagenome (Agilent, USA) to estimate the library size. Libraries were quantified using Qubit dsDNA HS kit on a Qubit 2.0 fluorometer (Life technologies, USA) and KAPA SYBR FAST qPCR Master mix and Illumina standards and primer premix (KAPA Biosystems, USA) as per the Illumina suggested protocol. Equal concentration of libraries was loaded on Illumina NextSeq 500 platform using NextSeq 500/550 v2 sequencing reagent kit (Illumina Inc., USA) and 150 bp paired-end sequencing of both types of libraries was performed at the Next-Generation Sequencing (NGS) Facility, IISER Bhopal, India.

### Analysis of amplicon reads

The amplicon reads were trimmed from the ends to remove ambiguous bases using NGSQC tool kit (Patel and Jain, 2012) and the reads with ≥3 ambiguous bases were removed. The paired-end reads were assembled together into single reads using FLASH (Magoc and Salzberg, 2011). The reads having ≥80% bases above Q30 were quality filtered and primer sequences were removed using cutadapt (Martin, 2011). The high-quality reads were then clustered and assigned by closed-reference OTU (Operational Taxonomic Unit) picking protocol (pick_closed_reference_otus.py) of QIIME v1.9 (Caporaso et al., 2010) using Greengenes database v13_5 (DeSantis et al., 2006) as a reference at ≥97% identity. The *de novo* OTU picking protocol (pick_otus.py) was adopted for the sequences that failed closed-reference OTU picking and were aligned against the Greengenes database using BLAT (Kent, 2002). The assignment of representative sequences from these OTUs was carried out by an in-house Perl script using Lowest Common Ancestor (LCA) approach (Chaudhary et al., 2015). The OTUs with low abundance (≤ 100 sequences) were filtered out to remove noise.

### Analysis of metagenomic reads

The whole genome shotgun reads were filtered using NGSQC tool kit by removing the reads with ambiguous bases and further selecting the reads having ≥80% bases above Q30. The high-quality metagenomic paired-end reads obtained from each sample were then assembled using MetaVelvet at a k-mer size of 77 bp (Namiki et al., 2012). The contigs obtained after *de novo* assembly were used for the prediction of open reading frames (ORFs) using MetaGeneMark (Zhu et al., 2010). The predicted genes obtained from all the samples were combined and clustered using cd-hit at 95% identity and 90% coverage length. To prepare a comprehensive dataset of metagenomic genes, the non-redundant gene repertoire generated from hot spring samples was combined with a non-redundant gene set from genes obtained from 2785 reference genomes from NCBI and genes from reference genomes in JGI genome portal (Grigoriev et al., 2012). The reads from each sample were mapped to this combined gene pool using Bowtie 2 (Langmead and Salzberg, 2012) for quantification of genes. The paired-end reads which mapped to the same gene, and cases where only one read from the paired-end read mapped on a gene and the other read remained unmapped, were considered and quantified. The genes having a total count of <10 were removed to avoid ambiguous genes in the analysis. The mapping of reads resulted in a total of 438,157 genes (count ≥ 10) including all samples, of which 355,675 genes were previously identified in assembled contigs from hot spring dataset, whereas 82,483 genes could not be predicted in assembled contigs and were identified through mapping of reads directly to the gene repertoire prepared using predicted genes from hot springs data, NCBI, and JGI genes. The genes were further annotated by alignment using BLAST 2.2.6 (Altschul et al., 1990) against eggNOG 4.0 (Powell et al., 2014), KEGG Database version 2011 (Kanehisa and Goto, 2000), and also by KEGG Automated Annotation Server (KAAS; Moriya et al., 2007). The information on pathway and KO was updated by retrieving the data from KEGG web-server in September 2015. The genes with best hits (bit-score ≥ 60 and *e* ≤ 10^−6^) were assigned with KO and eggNOG annotation, respectively, and were used for further analysis. The abundance of genes assigned to same KO IDs was summed and the relative abundance of each KO was calculated for each sample. Similarly, the abundance of genes assigned to same eggNOG IDs was summed and the relative abundance of each eggNOG was calculated for each sample.

Quantification of genomes was performed by mapping the reads to reference genomes using Bowtie 2 with default parameters. The reads which mapped to the reference genomes with ≥90% identity and with both the pairs mapping concordantly were considered as a hit. The abundance of identified genomes was further normalized by reference genome size and total number of reads in each sample (Gupta et al., 2016). To determine the taxonomic origin of metagenomic ORFs, the predicted ORFs were aligned using BLASTN against 2785 NCBI reference genomes. The hits were filtered using an *e*-value cut-off of ≤ 10^−5^ and alignment coverage ≥ 80% of the reference gene length. In the case of multiple best hits with equal identity and scores, LCA method was used for assignment. An identity threshold of 95% was used for assignment up to species level, 85% for genus level assignment and 65% for phylum level. A total of 1,53,098 genes (35%) could be assigned with taxonomy at least up to phylum level based on LCA algorithm.

### Statistical analysis

For amplicon reads, alpha diversity metrics including observed species and Shannon index were calculated at each rarefaction depth beginning from 100 sequences per sample to 2.2 million sequences per sample (*n* = 10 times) at a step size of 0.1 million using QIIME v1.9. Pielou's evenness was calculated using Vegan package in R to identify the distribution of species with respect to their proportion at each site (Oksanen et al., 2013). The abundance of various phyla and genera in samples was calculated and those which showed ≥1% abundance were plotted. Furthermore, the genera which had ≥1% abundance and showed significant (Wilcoxon Rank Sum test, *p* ≤ 0.05) difference in their abundance in two locations (Anhoni and Tattapani) were plotted. The taxonomic tree was constructed for genera having ≥1% abundance using GraPhlAn (Asnicar et al., 2015) to understand the taxonomic differences in different samples along with their abundances. Unweighted UniFrac distances were calculated on rarefied OTU table using QIIME v1.9 and were plotted on PCA (Principal Component Analysis) plot. To identify the discriminatory taxa based on the two locations, Random Forest analysis was carried out using randomForest package in R (Liaw and Wiener, 2002). In order to identify changes in diversity due to differences in temperature, multiple comparisons using Tukey's test were performed for number of observed species across the three groups of samples, i.e., moderately high temperature (40–55°C), high temperature (55–75°C) and extremely high temperature (≥75°C).

The relative abundance of KOs and eggNOGs calculated in each sample from individual metagenomic reads was used for further statistical analysis. Hellinger distances were calculated to estimate the beta diversity between samples. A total of 4749 KOs (with ≥10 counts) were used for further analysis and the odds ratio was calculated for a comparison between the two sites. Pathway analysis was performed using Reporter Features algorithm (Oliveira et al., 2008) to identify the pathways significantly enriched at both the locations due to temperature and other factors. The species identified from metagenomes were clustered using hierarchical clustering algorithm based on their proportions in each sample. The clustering pattern and species abundance (normalized) of each sample were plotted as a heatmap. The proportions of species were also correlated with temperature and those with significant Spearman's Rank correlation coefficient (FDR or False Discovery Rate adjusted *p* ≤ 0.05) and higher correlations (ρ ≥ 0.7 or ≤ −0.7) were plotted. Moreover, to find associations of species with temperature groups (moderately high, high, and extremely high temperature) on ordinations, PCA analysis was performed using Biplots with species plotted as vectors in ordinations.

### Nucleotide sequence data deposition

The nucleotide paired-end sequences have been deposited in NCBI SRA database with accession ids: SRR3961733, SRR3961734, SRR3961739, SRR3961740, SRR3961741, SRR3961742, and SRR3961743 for Whole Genome Sequencing (WGS) reads, and SRR3961735, SRR3961736, SRR3961737, SRR3961738, SRR3961744, SRR3961745, and SRR3961746 for 16S rRNA (V3 region) amplicon reads under the BioProject PRJNA335670.

## Results

### Physico-chemical analysis of the sampling sites

The temperature at the Anhoni hot springs ranged between 43.5 and 55°C, with pH-values between 7.5 and 7.8 at the three sites (Table 1). Tattapani hot springs had a much higher temperature than Anhoni and ranged between 61.5 and 98°C, with pH-values between 7 and 7.8 at the four different hot spring sites. The total dissolved solids (measured in ppm) varied between 590 to 690 at Anhoni and 600 to 880 at Tattapani. All these measurements were carried out on-site. The Anhoni hot spring samples showed high concentrations of Co, La, Fe, Hg, and Si (Supplementary Table 1 and Supplementary Figure 2). However, Pb, Zn, Ni, and B were observed to be high in Tattapani samples, indicating high levels of heavy metals in that site. Hg and S were observed in high concentration in the Anhoni samples as compared to Tattapani samples.

**Figure 1:**
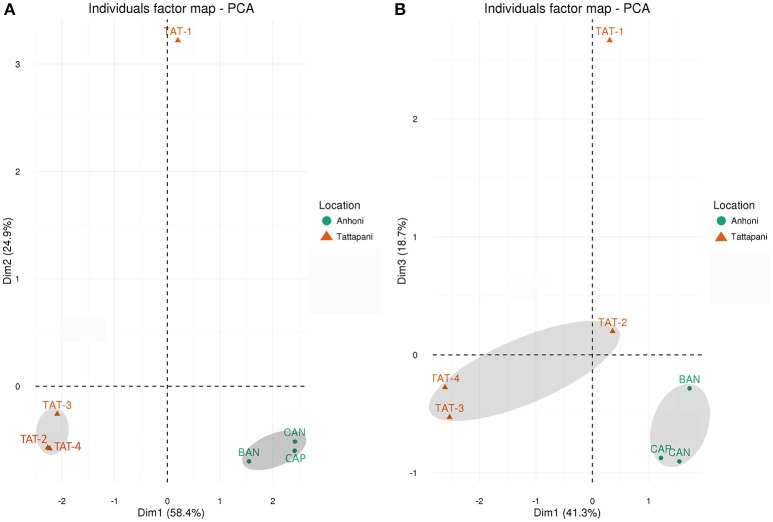
**PCA plots based on 16S rRNA and WGS reads. (A)** Unweighted UniFrac distances calculated using amplicon reads and plotted on PCA plot. **(B)** Hellinger distances calculated using KO abundances from metagenomic reads and plotted on PCA plot. All Anhoni samples clustered together, whereas the TAT-1 was found distant from other Tattapani samples.

### Amplicon and metagenomic analysis

A total of 21,881,886 high quality reads from 16S rRNA V3 region obtained from seven samples, each consisting of 2.3–4 million reads, were analyzed (Supplementary Table 2). A total of 2196 OTUs were obtained on closed-reference OTU picking. Since 5,242,917 reads (24% of sequences) could not be clustered and assigned using closed-reference OTU picking method, *de novo* OTU picking was performed for the remaining reads followed by taxonomic assignment. A total of 4018 OTUs (clustered at ≥97% identity) were obtained using both closed reference and *de novo* OTU picking methods, of which 90 OTUs (0.3% of total sequences) could not be assigned at any taxonomic level. A total of 99,156,908 high-quality metagenomic reads [14,165,272 ± 3,481,846 (mean ± *SD*)] were obtained from the seven samples. The assembly of reads resulted into 373,793 contigs (mean = 53,399; Supplementary Table 3) and a total of 438,157 genes (count ≥ 10) were identified in all samples. The gene repertoire from all hot spring samples was classified into KEGG Orthologous groups and eggNOG Orthologous groups. A total of 308,855 (70.49%) genes were annotated with 4749 KO IDs, and 313,798 (71.6%) genes were annotated with 31,794 eggNOG IDs.

### Hot springs at higher temperatures displayed lower microbial diversity and gene content

Alpha diversity analysis using Observed Species, Shannon Diversity Index, and Pielou's evenness was carried out to determine the species richness and evenness in the samples. TAT-1 sample having the highest temperature among all samples showed the lowest species richness and diversity as compared to all other sites at each rarefying depth (Supplementary Figure 3). Beta diversity analysis was carried out using Unweighted UniFrac distances. Lower UniFrac distances and close clustering was observed among the Anhoni samples, and also among three of the four Tattapani samples on the PCA plot (Figure 1A). TAT-1 sample from Tattapani showed higher UniFrac distance from all other sites. The region-specific clustering of Anhoni and Tattapani samples on the PCA indicates phylogenetic similarity in samples obtained from a similar region. It also highlights the variation in microbial community of the two locations due to the differences in geographical regions and observed temperature.

Shannon diversity index was used to estimate the gene diversity within the samples and showed a significantly lower (*p* ≤ 0.05) gene diversity in Tattapani as compared to Anhoni (Supplementary Table 4). The Tattapani samples showed separate clustering from Anhoni samples using Hellinger Distances based on KO proportions and eggNOG proportions (Figure 1B and Supplementary Figure 4). The Tattapani samples showed higher Hellinger distances, which could be due to the wide temperature range. TAT-1, which has the highest temperature showed the largest distance from other samples collected from the same geographical location. This suggests that temperature might be playing a significant role in determining the metabolic potential of the microbial community in this region. Tukey's test showed a significant reduction (*p* ≤ 0.001) in the number of observed species at an extremely high temperature (≥75°C) compared to other two groups of moderately high (40–55°C) and high temperature (55–75°C; Supplementary Figure 5).

### Microbial community structure

**Figure 2:**
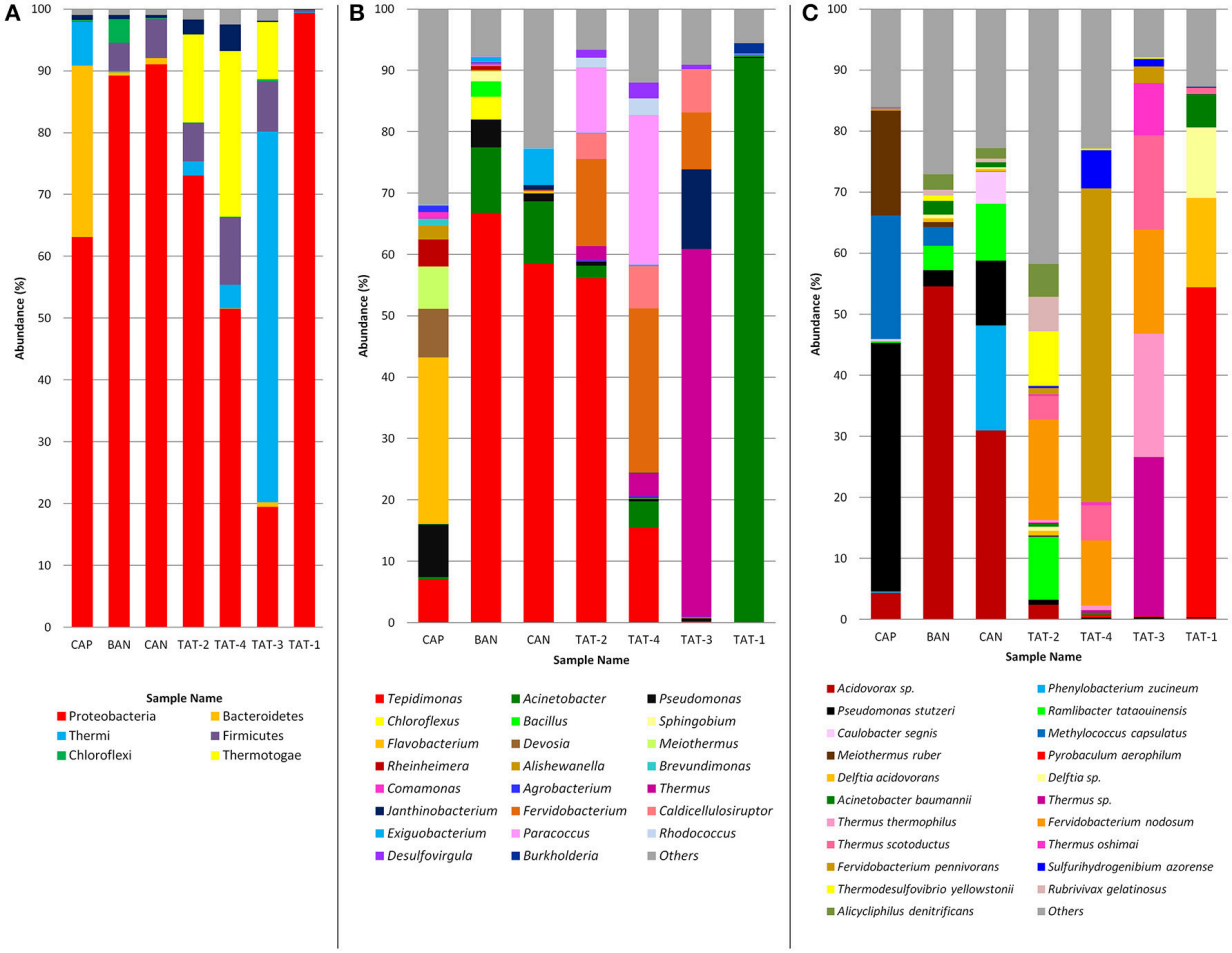
**Relative abundance of microbial community at different taxonomic levels. (A)** Phylum abundance in hot springs samples. Phylum abundance ≥ 1% are plotted in the bar plot. **(B)** Genus abundance in hot springs samples. Genus abundance ≥ 1% are plotted in the bar plot. **(C)** Species abundance in hot springs samples. Species abundance ≥ 5% are plotted in the bar plot. **(A,B)** are prepared from the results from 16S rRNA gene (V3 amplicon) sequencing and C is prepared from the taxonomic analysis of metagenomic reads.

Based on the amplicon analysis, Proteobacteria was found to be the most abundant (52–99%) phyla in all samples, except TAT-3 (19.5%) which had the highest abundance (59.97%) of phylum Thermi (Figure 2A). Thermotogae was also among the abundant phyla in TAT-2 (14.2%), TAT-3 (9.2%), and TAT-4 (26.8%). Firmicutes were found abundant in all samples (~4–10%), except CAP and TAT-1. At the genus level, *Tepidimonas* was the most abundant genus in BAN, CAN, and TAT-2 (56–67%) and was also abundant in TAT-4 (15.4%; Figure 2B). However, *Flavobacterium* was the most abundant in CAP (27.1%), *Thermus* in TAT-3 (60%) and *Acinetobacter* in TAT-1 (92.1%). TAT-4 also showed a higher abundance of *Fervidobacterium* (26.8%) and *Paracoccus* (24.4%). *Pseudomonas* was abundant in Anhoni samples and was noticeably high in CAP (8.5%). Species identification was carried out using WGS reads, and the species with ≥5% abundance were plotted in bar plots (Figure 2C). A higher abundance of *Acidovorax* sp. was observed in BAN (54.6%) and CAN (30.9%) samples, and *Pseudomonas stutzeri* was abundant in CAP (40.8%). An archaeal species, *Pyrobaculum aerophilum* was abundant (54.0%) in the TAT-1 sample. Two *Fervidobacterium* species, *Fervidobacterium pennivorans* (51.4% in TAT-4) and *Fervidobacterium nodosum* (16.5% in TAT-2; 17.1% in TAT-3 and 10.9% in TAT-4), were observed as abundant in other Tattapani samples. Three known *Thermus* species, *Thermus thermophilus* (20.2%), *Thermus scotoductus* (15.5%), and *Thermus oshimai* (8.6%) and an unknown Thermus species (26.1%) were found abundant in TAT-3. *Ramlibacter tataouinensis* was also among the abundant species in TAT-2, CAN, and BAN. This species is a cyst-producing aerobic chemoautotroph and is commonly found to be associated with dry environments (Heulin et al., 2003). The presence of this soil-dwelling bacterium in a hot spring water sample is intriguing.

**Figure 3:**
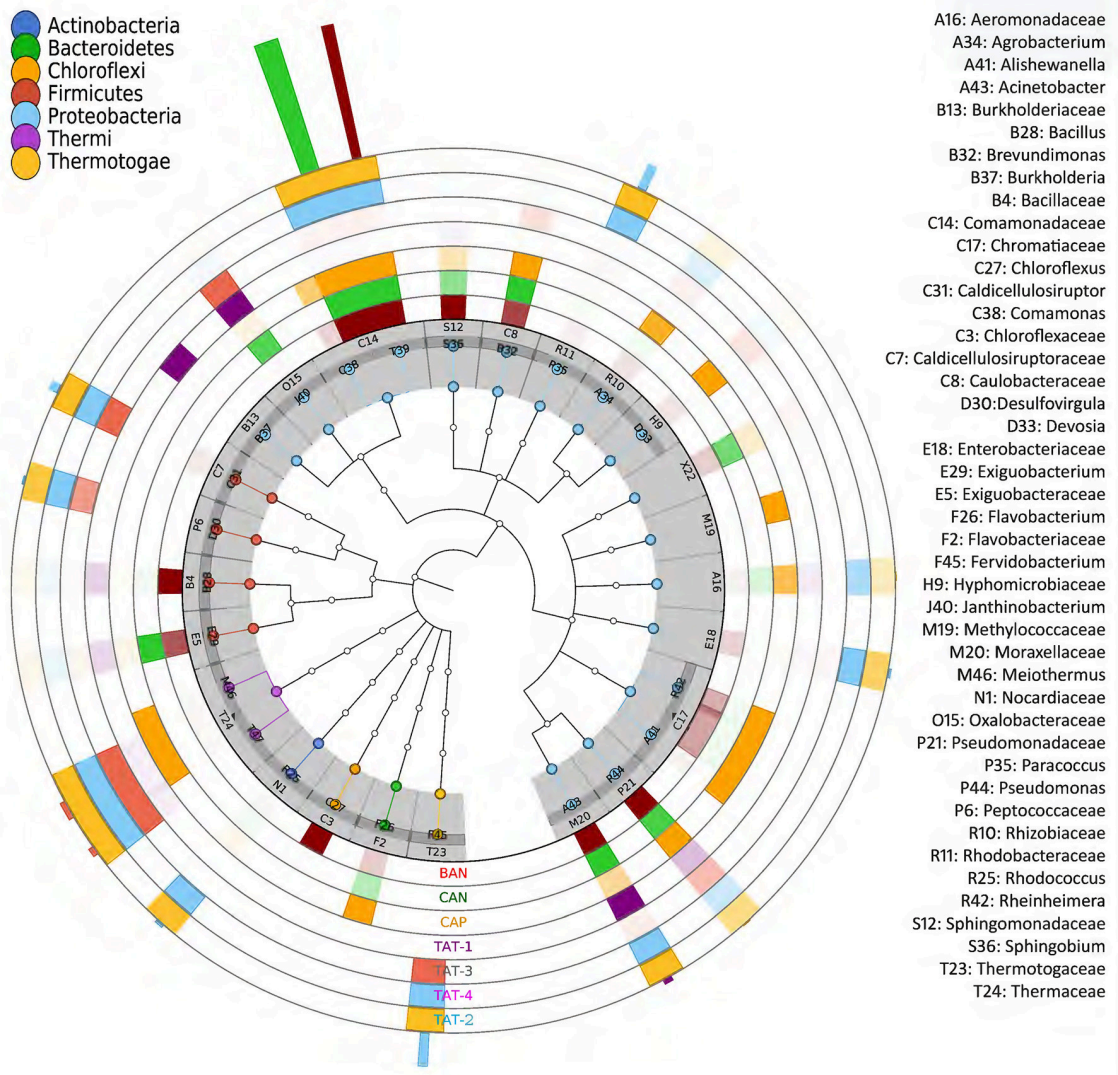
**Taxonomic tree showing microbial community structure in hot spring samples**. The families having ≥ 1% abundance are plotted. The outer seven concentric circles depict a heatmap based on the taxonomic abundances in different samples. The color of outermost bars depicts the sample in which the taxonomic group is abundant. The color in the inner nodes depicts its phylum.

Among the Anhoni samples, BAN and CAN showed similar taxonomic profiles, whereas CAP displayed a different taxonomic composition. Similarly, TAT-2 and TAT-4 from Tattapani displayed similar taxonomic profile, whereas TAT-1and TAT-3 had a strikingly different taxonomic structure (Figure 3). *Desulfovirgula, Fervidobacterium*, and *Thermus* were found to be significantly associated (*p* ≤ 0.05) with Tattapani, whereas *Flavobacterium, Pseudomonas*, and *Rheinheimera* were found to be significantly associated (*p* ≤ 0.05) with Anhoni (Supplementary Figure 6).

**Figure 4:**
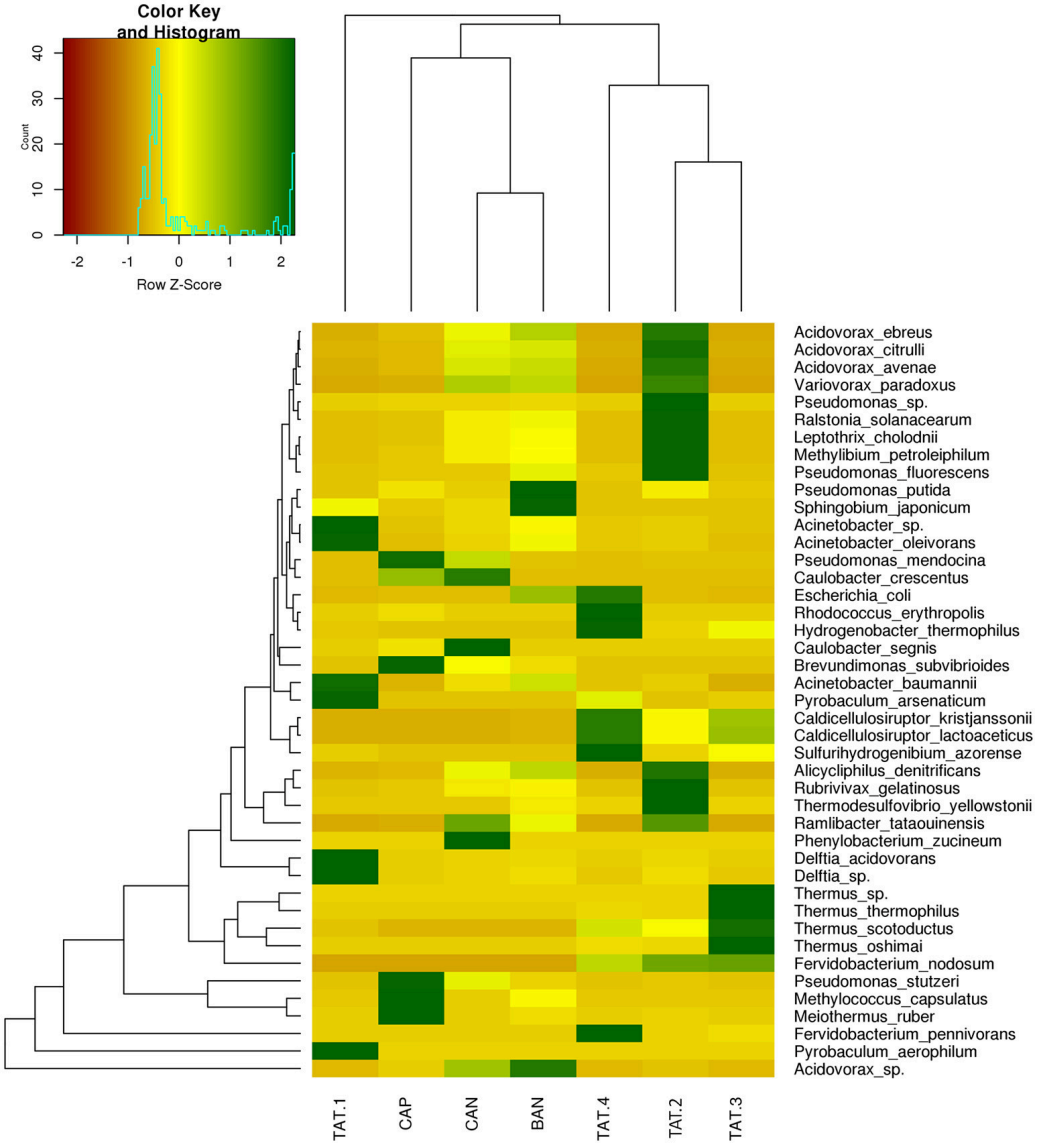
**Heatmap showing relative abundance of species**. Species with ≥ 5% abundance in each WGS sample are plotted on heatmap. The dendrograms show hierarchical clustering between species and samples.

**Figure 5:**
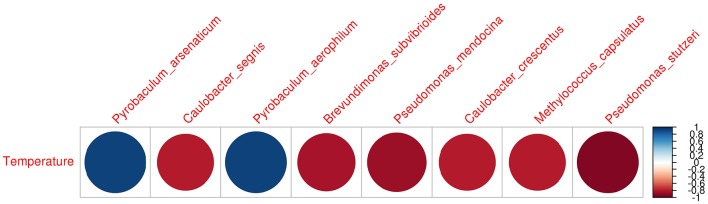
**Spearman's Rank correlation of species with temperature**. Species proportions obtained from metagenomic analysis were correlated with temperature and those with significant Spearman's Rank correlation coefficient (FDR adjusted *p* ≤ 0.05) and higher correlations (ρ ≥ 0.7; ρ ≤ −0.7) were plotted.

**Figure 6:**
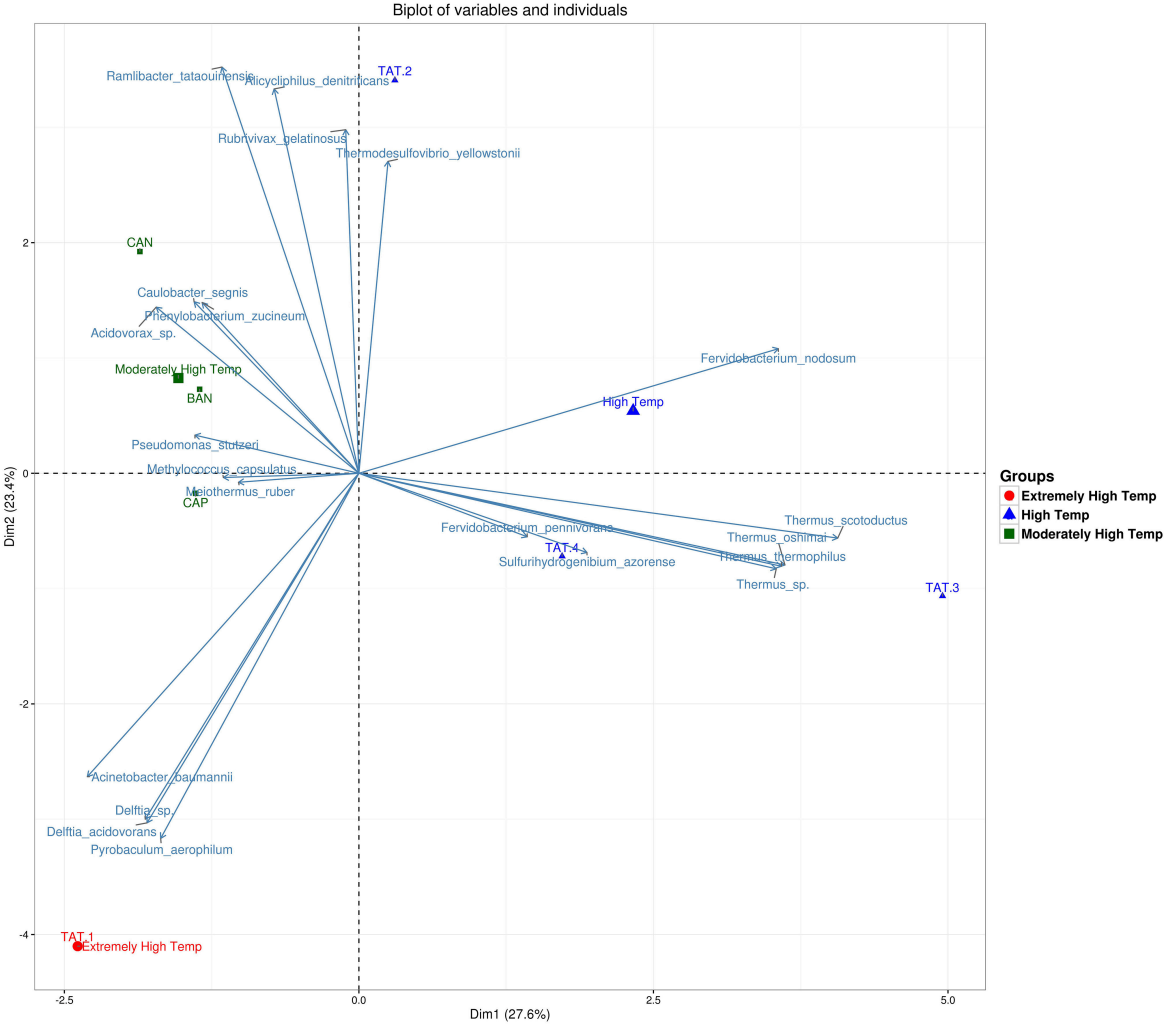
**Biplot showing an association of species with sample groups based on temperature**. Principle component analysis of samples grouped using temperatures and their association with species are shown as vectors on ordinations.

The heatmap depicting the hierarchical clustering of species and samples using species proportions at each site (Figure 4) indicated the close clustering of three samples from Tattapani (TAT-2, TAT-3, and TAT-4), and similarly, the three samples from Anhoni (BAN, CAN, and CAP) were found to cluster separately. Interestingly, TAT-1 was observed to be the farthest from all samples in the dendrogram suggesting the existence of a unique microbial population enriched in archaeal species in this region. In order to find associations of species with temperature, the Spearman's rank correlation coefficient was calculated for each species with temperature and those with significant correlation coefficients (FDR adjusted *p* ≤ 0.05; ρ ≥ 0.7 or ≤ −0.7) were plotted (Figure 5). *Pyrobaculum aerophlilum* and *Pyrobaculum arseniticum* correlated positively with temperature, whereas *P. stutzeri, Methylococcus capsulatus*, and *Caulobacter* spp. correlated negatively showing their association with lower temperature sites. The association of *P. aerophilum* and *Delftia* species was observed with extremely high temperature, and *Themus thermophilus* and other thermophilic species were found to be associated with high temperature when plotted in ordinations using Biplot (Figure 6). A clear distinction was also observed with *M. capsulatus* and *Meiothermus* showing association only with CAP, making this site different from other hot springs. Species association also varied between TAT-2, TAT-3, and TAT-4 suggesting unique species diversity at each site.

A total of 24 OTUs were observed as discriminatory among Anhoni and Tattapani samples using Random Forest analysis (Supplementary Table 5), out of which nine OTUs belonged to *Pseudomonas* (*P. stutzeri, P. alcaligenes*, and unknown species of *Pseudomonas*). OTUs belonging to genus *Pseudomonas, Acinetobacter, Exiguobacterium*, and *Vogesella* were discriminatory for Anhoni, whereas *Paenibacillus, Planomicrobium*, and *Devosia* were found as discriminatory genus for Tattapani.

### Identification of functions in Anhoni and Tattapani

The analysis of eggNOG classes revealed interesting trends with respect to the enrichment of functional categories (Supplementary Figure 7). The functions for inorganic ion transport, lipid metabolism, and secondary metabolites were enriched in Anhoni, whereas energy production, nucleotide metabolism, cell cycle control, replication, and post-translational modifications were enriched in Tattapani. It is apparent that the functions and pathways related to nucleotide metabolism, DNA replication and energy production were enriched (FDR adjusted *p* ≤ 0.05) in samples obtained from higher temperature hot springs (Tattapani).

**Figure 7:**
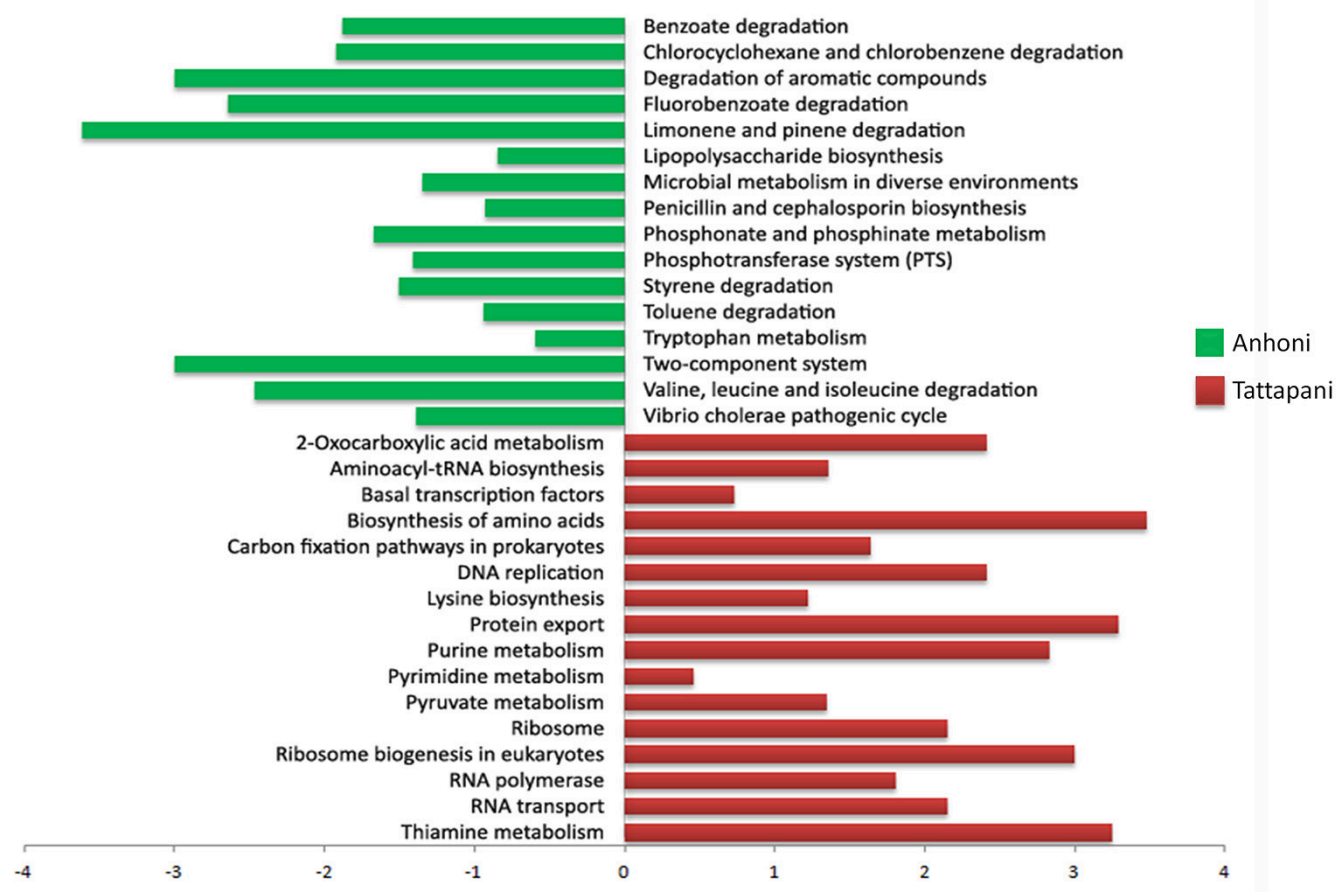
**Fisher's exact test for pathways in Anhoni and Tattapani samples**. The positive bars (in green) are pathways enriched in Anhoni samples, whereas the negative bars (in red) are pathways enriched in Tattapani samples.

A comparison of KEGG pathways using the Odds ratio for enrichment, and further by Fisher's exact test on the proportion of enriched KOs belonging to a pathway in the two locations revealed several major pathways significantly associated with Anhoni and Tattapani (Figure 7). Log odds ratios calculated for significantly discriminatory pathways through Fisher's exact test revealed pathways for degradation of hydrocarbons such as benzoate, toluene, xylene, fluorobenzoate, chlorocyclohexane, chlorobenzene, etc., as significantly (*p* ≤ 0.05) associated with Anhoni, whereas DNA replication, purine, and pyrimidine metabolism were significantly associated with Tattapani, which corroborates with the results of functional analysis using eggNOG classes for Tattapani.

### Enriched KEGG metabolic pathways in Tattapani

**Figure 8:**
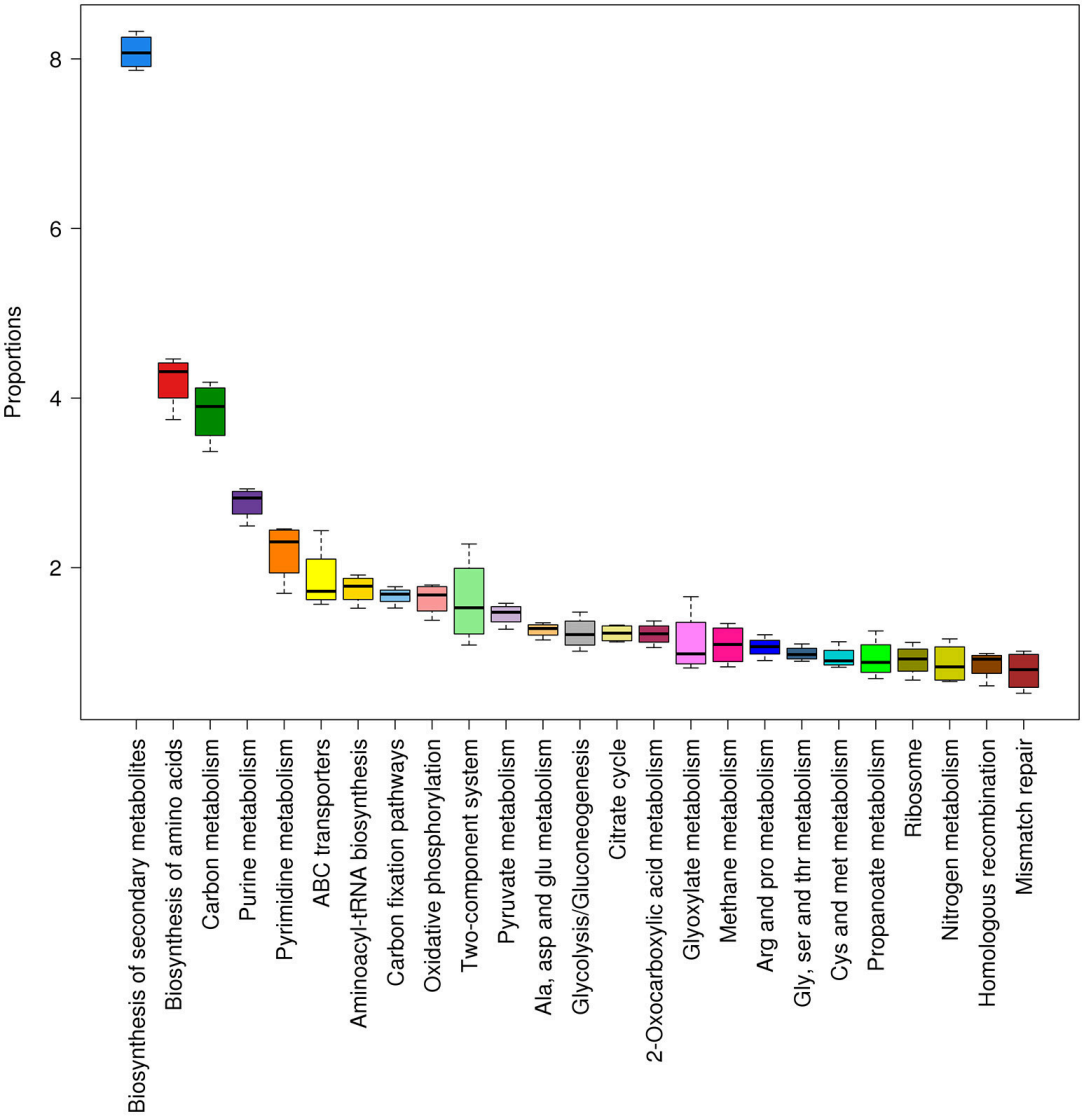
**Abundant KEGG pathways found in Tattapani samples**. The pathways and their proportions in Tattapani samples are shown using box plots (≥1% proportion). The samples showed a higher abundance of pathways for general cell functions and certain specific functions such as nitrogen metabolism and methane metabolism.

A detailed analysis of KEGG pathways in Tattapani showed that pathways for biosynthesis of secondary metabolites, amino acid biosynthesis, nitrogen metabolism, and other cellular functions were abundant in this location (Figure 8). The higher abundance of TCA cycle and oxidative phosphorylation pathways revealed by KEGG pathway analysis and abundance of energy production pathway revealed by eggNOG analysis shows that aerobic respiration is the major mechanism for energy generation by the microbial community at this site. The high abundance of *P. aerophilum* in TAT-1 (Figure 2C) and its earlier reported nitrate reducing properties (Afshar et al., 2001) corroborates with the observed nitrogen metabolism pathway at this site.

### Degradation of hydrocarbons in Anhoni

**Figure 9:**
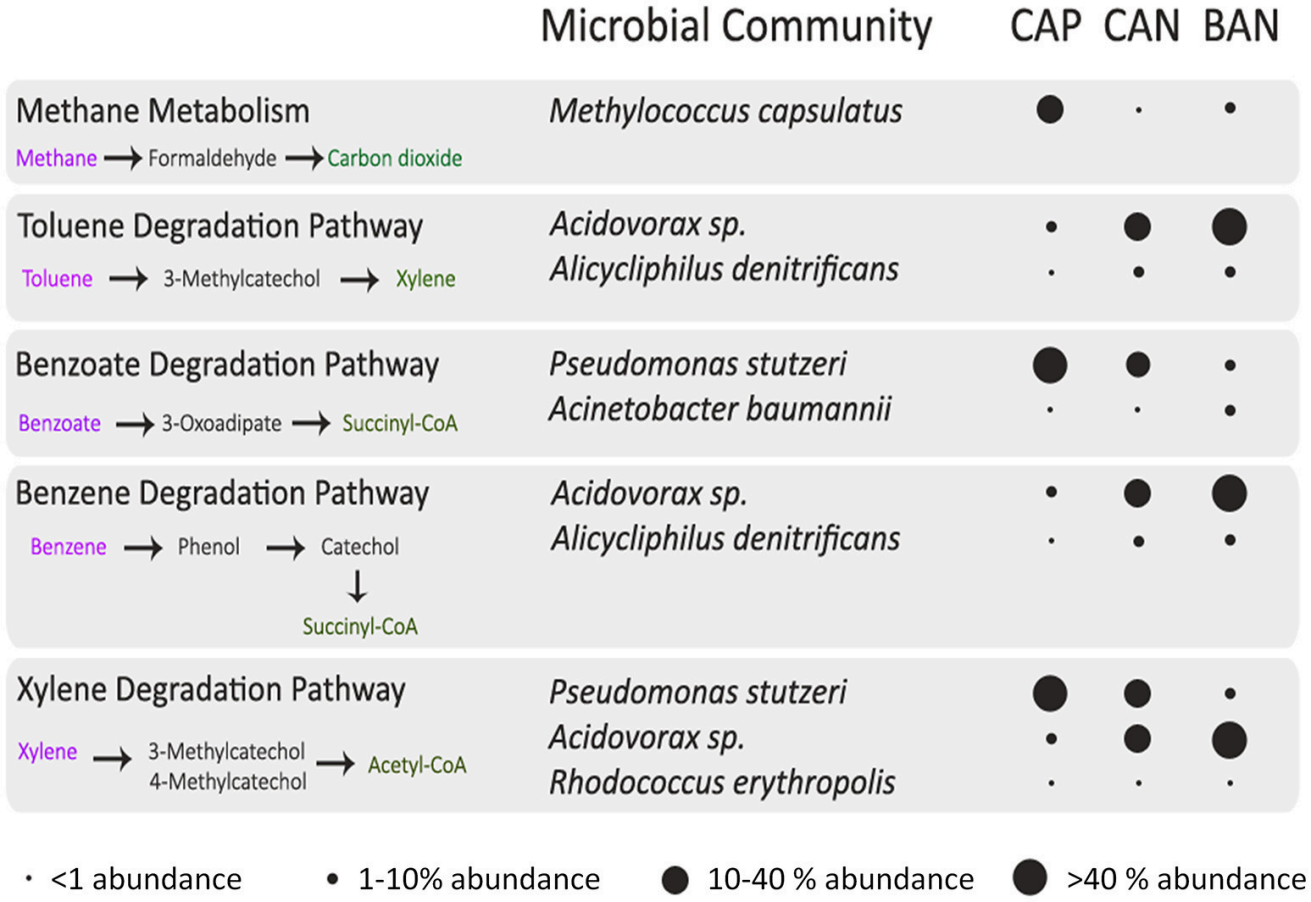
**Microbes associated with hydrocarbon degradation pathways and their abundances in Anhoni samples**. The major intermediates and the microbes involved in the degradation pathway are described in this figure. The diameter of the circle represents the relative abundance of microbes in the three Anhoni samples.

One of the interesting findings of this study is the presence of hydrocarbon degradation pathways in Anhoni Samples. We performed a comprehensive analysis of these pathways to identify the various intermediates and end products, microbes harboring these pathways and their relative abundance in the CAP, BAN, and CAN samples which are summarized in Figure 9. Methane metabolism was found to be highly enriched in CAP which is reported to have a high proportion (>80%) of methane in previous studies (Sarolkar, 2015). The high (20.27%) abundance of *M. capsulatus* which has the ability to utilize methane as a source of energy corroborates with the enrichment of methane metabolism pathway at this site (Ward et al., 2004). The genes involved in methane metabolism were present in the metagenomic dataset and could be annotated with this genome by taxonomic assignment (Supplementary Figure 8). Oxidation of methane to methanol and subsequently to formaldehyde is performed by this microbe which is further utilized in other downstream pathways including conversion to Acetyl-CoA for energy production.

Complete metabolic pathways for the degradation of toluene, xylene, benzoate and benzene were found in various microbes identified in Anhoni samples (Supplementary Figures 9–11). Enzymes for toluene degradation pathway were found as most abundant in BAN and were also present in CAP and CAN samples, contributed mainly by *Acidovorax* sp. and *Alicycliphilus denitrificans* (Figure 9). The enzymes for benzoate and xylene degradation, which are downstream pathways of toluene degradation, were higher in CAP as compared to BAN and CAN samples and were predominantly contributed by *P. stutzeri* and *Acinetobacter baumannii*. Benzene on conversion to phenol and subsequently to catechol can be degraded via benzoate degradation pathway and the enzymes for this pathway were mainly contributed by *Acidovorax* sp. and *Alicycliphilus denitrificans*. Xylene degradation pathway was also found to be abundant in Anhoni samples and was contributed by microbial community mainly comprising of *P. stutzeri, Acidovorax* sp., and *Rhodococcus erythropolis*. It is interesting to note that most of the above microbes involved in the metabolism of complex hydrocarbons do not possess all enzymes required in the respective pathway. However, together as a community, they could complete the set of enzymes of a pathway, and thus, achieve the ability to carry out complex metabolism. For example, in the case of benzoate degradation pathway in CAP, 3-oxoadipate CoA-transferase enzyme was not present in *P. stutzeri*, whereas all other seven enzymes (out of the total eight enzymes) involved in the pathway were present. Another microorganism, *Acidovorax* sp., which was abundant in the same microbial community is found to harbor this enzyme and perhaps contribute toward the completion of the pathway (Supplementary Figure 12). Similarly, in xylene degradation pathway, individual microbes including unknown microbial species contribute different steps in xylene degradation and complete the pathway as a community. Taken together, it is apparent that Anhoni samples possess a unique microbial community with the ability to utilize various hydrocarbons as a source of energy via aerobic respiration. By mapping these enzymes to their respective pathways, it was observed that benzoate and xylene are utilized and converted to Succinyl-CoA and Acetyl-CoA, respectively, which then enters the TCA cycle to generate energy (Supplementary Figure 13).

## Discussion

The hot springs of Anhoni and Tattapani are located in the margins of Gondwana coalfields of India and have been reported as important geothermal resources (Pandey and Negi, 1995). The presence of volcanic tuffs and interlayer basic silts, which are flat intrusions of igneous rocks formed between the pre-existing layers of rocks, has also been reported under these regions. The volcanic sediments are known to constitute hydrocarbons and other organic compounds and also release organic gases (Farooqui et al., 2009). The long-term seepage of hydrocarbons, either as macroseepage or as microseepage can bring about a diverse array of mineralogical and chemical changes that are favored by the development of near-surface oxidation or reduction zones (Khan and Jacobson, 2008). Surface geochemical methods are based on the premise that hydrocarbon gas components migrate from sub-surface petroleum reserves through faults and fractures and leave their signatures in near surface soils (Price, 1986; Tedesco, 2012). Petroleum microseepage in the soil causes several chemical reactions modifying the oxidation-reduction potential of the soil which plays an important role in the mobility of elements. It can be inferred that in Anhoni, a lot of igneous bodies cut the sedimentary bodies and water present deep along these joints gets heated from the mantle thus, finding space to come to the surface. As they travel up and cool they start to deposit the elements with lower solubility i.e., the hydrocarbons, progressively. The same water continues to cycle in this process and brings elements associated with hot springs from the mantle to the crust, making it enriched in hydrocarbons and other inflammable compounds.

Anomalous amounts of vanadium, chromium, nickel, cobalt, manganese, mercury, copper, molybdenum, uranium, zinc, lead, and zirconium are positive indicators of petroleum deposits (Duchscherer, 1983). The migrating hydrocarbons create a reducing environment in the soil and subsurface, which increases the solubility of many trace and major elements (Mongenot et al., 1996). The samples from Anhoni have anomalously high concentrations of V (120.42–267.59 ppb), Cr (53.77–189.14 ppb), Co (733.48–2882.70), Mn (83.51–255.74 ppb), Hg (333.00–555.00 ppb), Cu (102.9–276.67), and Mo (72.55–171.66) compared to regions (Bowen, 1979). This strongly indicates the presence of microseepage of petroleum deposits through the Narmada-Son lineament fracture zone into the near surface soils at Anhoni. However, this hypothesis needs further studies, which is beyond the scope of the current study.

The Tattapani geothermal field at Chhattisgarh consists of several hot springs having a broad temperature range from 52 to 98°C and spread over an area of around 0.5 km^2^ (Sarolkar, 2010). The temperature of different reservoirs at this site has been reported to be as high as 230 ± 40°C at a depth of 2 km and 112 ± 30°C at a depth of 1 km (Vaidya et al., 2015). In another study, the chemical analysis of water from this site was found to contain moderate chloride, sulfate, silica, and sodium content, followed by low potassium, calcium, and arsenic content (Sarolkar and Das, 2006). This geothermal site has been reported majorly for its extremely high temperature, due to which a lower microbial diversity and a distinct functional profile was observed in the results of this study.

Phylum abundance results obtained from the amplicon sequencing data showed phylum Proteobacteria to be abundant in almost all the samples. Proteobacteria has also been reported from many studies based on the 16S rRNA analysis of hot springs with moderately high and very high temperatures (44–110°C) at various geographical locations, including India (Bowen De Leon et al., 2013; Chan et al., 2015; Ghelani et al., 2015). Since, Proteobacteria have been found in other hot spring studies including Indian hot springs, and was also one of the abundant taxa in this study, it appears to be indigenous to this region. Other phyla, such as Thermi and Thermotogae were also found abundant at both the sites. Phylum Thermotogae observed as abundant in Tattapani is found associated with hot springs of high temperature and is reported majorly for its high heat tolerance (Chan et al., 2015; Kanoksilapatham et al., 2015). *Tepidimonas* sp., belonging to phylum Proteobacteria observed as abundant in BAN, CAN, TAT-2, TAT-4, and CAP samples, has been isolated and sequenced from Chhoti Anhoni in an earlier study (Dhakan et al., 2016). The draft genome construction and analysis of the *Tepidomonas taiwanensis* genome revealed the presence of genes for sulfur metabolism, ammonia metabolism, nitrogen fixation, assimilation of organic acids, and a large variety of proteases. *Gulbenkiania mobilis*, another Proteobacteria was also cultured from the same environment and its first draft genome was reported in another study (Saxena et al., 2015). Though culturable, this genome was not among the most abundant genomes found in this study. The genome analysis of *Gulbenkiania mobilis* revealed its unique sulfur-metabolizing properties.

The metagenomic analysis of Anhoni region revealed enrichment of hydrocarbon degrading microbes, enzymes, and pathways. The presence of pathways such as benzoate, toluene, and xylene degradation at Anhoni, specifically in CAP sample, underscores the potential of the inherent microbial community to metabolize hydrocarbons as a source of energy. *P. stutzeri* and *Acidovorax* sp. which are known to harbor the enzymes for hydrocarbon degradation were found abundant at Anhoni. *P. stutzeri* is among the important alkane-degrading microorganisms reported for the bioremediation of crude oil, oil derivatives and aliphatic hydrocarbons (Lalucat et al., 2006). Potential degradation of toxic hydrocarbons such as, benzene, xylene and benzoate by *P. stutzeri* are well-reported in the literature and is also observed in the results of this study. *Acidovorax* sp. which was among the abundant species in CAP is also reported for its hydrocarbon degrading properties at high temperatures (Singleton et al., 2009). Microbes belonging to phylum Proteobacteria found abundant at this site are known to be highly abundant in petroleum-contaminated terrestrial and aquatic environments (Kimes et al., 2013; Bao et al., 2016). A 16S rRNA-based study from a hydrothermal vent (270–325°C) with similar geochemistry at the Gulf of California, where the organic matter in the sediments are converted to aliphatic and aromatic hydrocarbons under high temperature, suggested a resident sulfate-reducing bacterial population, such as *Desulfobacter, Desulforhabdus, Thermodesulforhabdus*, etc. (Dhillon et al., 2003).

The known high percentage of methane gases at Anhoni corroborates well with the presence of methanotrophic species such as *M. capsulatus* (Sarolkar, 2015). The higher abundance of this pathway, particularly in CAP, shows the importance of this site for the isolation of novel methanotrophic species. *M. capsulatus* is known to convert formaldehyde through Ribulose-P pathway to form Acetyl-CoA for subsequent energy generation pathways which were evident in our data (Supplementary Figure 8; Ward et al., 2004). Our data also suggests the conversion of formaldehyde to formate which then enters the Acetyl-CoA pathway. However, this pathway is not reported to be carried out by *M. capsulatus* (Ward et al., 2004), indicating the presence of other methanotrophs in CAP. *Meiothermus ruber*, another abundant species in CAP is a thermophile observed to be associated with moderately high temperature (Tindall et al., 2010). Methane-producing and sulfur-utilizing thermophilic bacteria belonging to the genus, such as *Thermococcus, Acinetobacter, Pseudomonas*, and *Methylobacterium* have also been reported to be associated with high-temperature petroleum reservoirs (50–120°C) in California (Orphan et al., 2000).

One of the interesting insights from this study is that all enzymes involved in a particular hydrocarbon degradation pathway were not found in a single microbial species; however, as a microbial community, the entire set of genes for the degradation of that hydrocarbon could be completed. Furthermore, the results also hint toward the existence of novel species in these hot springs which might harbor the complete degradation pathways and can be confirmed by their isolation and sequencing, therefore, providing leads for further studies. The species and enzymes identified in this study and future studies from this site could be used as promising bioremediation agents in oil spills and other hydrocarbon-contaminated regions (Kimes et al., 2013; Gaur et al., 2014).

Tattapani hot spring having an extremely high temperature (61.5–98°C) displayed a diverse thermophilic population and their pathway analysis suggested the functional adaptations of these thermophiles to survive at high temperatures. Extreme environments such as hot springs with very high temperature are commonly found to be inhabited by chemolithoautotrophic thermophilic species majorly from archaebacteria, and from Aquifex and Thermotoga phyla in eubacteria (Stetter, 1996, 1999). The high abundance of *P. aerophilum* in this environment is an interesting finding as it is a unique hyperthermophilic archaeal species reported extensively for its nitrate reducing properties with the help of a molybdenum-associated nitrate reductase which is distinct from other mesophilic nitrate reductases (Afshar et al., 2001). *P. aerophilum* also found to be abundant in Manikaran hot spring in India (96°C), is also known to grow anaerobically by dissimilatory nitrate reduction, which is an exclusive catabolic feature of this species (Afshar et al., 2001; Bhatia et al., 2015). *Fervidobacterium thermophilum* and *Fervidobacterium pennivorans* are among the other thermophilic eubacteria observed to be abundant in Tattapani, and are reported to produce thermostable cellulases and keratinases (fervidolysin) respectively, which have potential application in the conversion of biomass to biofuels at high temperature and other biotechnological processes (Kim et al., 2004; Wang et al., 2010). Overall, a high species diversity was observed at the sites with moderately high temperature (40–55°C) i.e., from the Anhoni site, compared to the site at extremely high temperature i.e., TAT-1 (98°C; Supplementary Figure 5). The presence of hydrocarbons at Anhoni site could be a contributing factor for the increase in species diversity at this site, as it provides a rich carbon source to the microorganisms thus, helping them to survive despite high temperature (Head et al., 2006). Large community diversity is generally observed in petroleum or hydrocarbon-contaminated environments and is also observed in the Anhoni samples (Kimes et al., 2013; Bao et al., 2016).

The presence of genes for replication and DNA repair pathways appear to be important in Tattapani microbial community to survive in adverse physical conditions such as, high temperature, which cause considerable damage to DNA. The enrichment of genes associated with DNA repair systems and homologous recombination have been well-reported in extreme environments (Xie et al., 2011; Jimenez et al., 2012). Some recent studies have shown that the evolutionary rate of microbial communities is governed by the environmental conditions (Gupta and Sharma, 2015), and microbes in extreme habitats evolve faster with extensive DNA repair system and high mutation rates to cope with the deleterious effects of environment on their genomes as compared to those in stable environments (Li et al., 2014). Despite having a higher abundance of pathways such as replication and nucleotide metabolism, lower species richness was observed in Tattapani which indicates that very few microbial species are able to adapt and survive in extreme conditions.

The present metagenomic exploration of the hot springs located in central India is perhaps the largest comprehensive metagenomic study of any geographical location in India including hot springs, carried out using both 16S rRNA amplicon and shotgun sequencing. It has provided novel insights into the taxonomic, functional, and metabolic diversity of the unique microbial community surviving in these hot springs. The presence of chemoorganotrophic thermophiles degrading complex hydrocarbons at the Anhoni hot springs and extensive survival mechanisms at the Tattapani hot springs are novel revelations of the study. The present analysis is however limited by the availability of known species of microbes in reference datasets and opens up new opportunities to decipher the genomes of yet unknown microorganisms in these hot springs.

## Author contributions

VS and RS conceived the idea. RS and PW designed and performed the experiments and sequencing. AC and AG carried out the elemental analysis and interpreted its results. DD and PM performed the computational analysis. RS, DD, PM, AG, and VS analyzed the data and interpreted the results. RS, DD, and PM prepared all the figures and tables. RS, DD, PM, AG, and VS wrote and reviewed the manuscript.

### Conflict of interest statement

The authors declare that the research was conducted in the absence of any commercial or financial relationships that could be construed as a potential conflict of interest.

## Supplementary material

Supplementary Figure 1 **Geographical location and photographs of sampling sites of Anhoni and Tattapani hot springs**. The given map was retrieved from Google Maps (2016).

Supplementary Figure 2 **Graphical representation of elemental composition at the seven sample sites**.

Supplementary Figure 3 **Alpha diversity in the Hot spring samples. (A)** Shannon index and **(B)** Observed Species were calculated by rarefying from 100 to 2.2 million sequences at a step size of 0.1 million. This analysis was carried out using amplicon reads.

Supplementary Figure 4 **PCA plot prepared using Hellinger distances based on eggNOG proportions**.

Supplementary Figure 5 **Multiple comparisons of number of observed species with moderately high, high and extremely high-temperature locations**. Multiple comparisons using Tukey's test were performed between samples grouped on the basis of temperature (moderately high, high, and extremely high). The mean ± *SD* are plotted with significant variations shown as ^***^*p* ≤ 0.001.

Supplementary Figure 6 **Significantly discriminatory genera (***p*** ≤ 0.05) in Anhoni and Tattapani sites**.

Supplementary Figure 7 **eggNOG functional classes and their distributions in the two sites**. The box plot shows the abundance of eggNOG functional categories in Anhoni and Tattapani.

Supplementary Figure 8 **A pathway map of methane metabolism showing the observed KOs (highlighted in red) in this dataset**. Methane is oxidized to methanol via methane monooxygenase and converted to formaldehyde with the help of methanol dehydrogenase. The KOs which are commonly present in all three sites (CAP, CAN, and BAN) of the Anhoni region are shown.

Supplementary Figure 9 **A pathway map of toluene degradation showing the observed KOs (highlighted in red) in this dataset**. Toluene is degraded to Benzoate and 3-methylcatechol which enters the benzoate degradation and xylene degradation pathways respectively for further downstream processes. The KOs which are commonly present in all three sites (CAP, CAN, and BAN) of the Anhoni region are shown.

Supplementary Figure 10 **A pathway map of xylene degradation showing the KOs (highlighted in red) observed in our dataset**. Xylene is degraded and converted to Acetyl-CoA by the community microbes and enters the TCA cycle for energy generation. The KOs which are commonly present in all three sites (CAP, CAN, and BAN) of the Anhoni region are shown.

Supplementary Figure 11 **A pathway map of benzoate degradation showing the observed KOs (highlighted in red) in this dataset**. Benzoate is degraded and converted to intermediates such as Pyruvate and Succinyl-CoA, finally entering into TCA cycle for energy generation. The KOs which are commonly present in all three sites (CAP, CAN, and BAN) of the Anhoni region are shown.

Supplementary Figure 12 **Bar charts showing the proportion of key enzymes of benzoate pathway observed in this dataset along with their taxonomic annotations**.

Supplementary Figure 13 **Utilization of hydrocarbons by the microbial community for generation of energy**. The hydrocarbons are utilized as a substrate by the chemoorganotrophic thermophiles to produce energy. The degradation pathways which were observed to be completely present in Anhoni samples are shown with some of the intermediates and their end-products.

Supplementary Table 1 **Elemental analysis of the hot spring samples**.

Supplementary Table 2 **Number of 16S rRNA (V3 hypervariable region) amplicon reads obtained per sample**.

Supplementary Table 3 **Sequencing and assembly statistics of the hot spring samples obtained after ***de novo*** assembly**. The size of contigs ranged from 1817 to 189,789 bp indicating a taxonomic diversity across different samples.

Supplementary Table 4 **The alpha diversity metrics calculated for 16SrRNA and metagenomics reads are shown for both sites (mean ± ***SD***)**.

Supplementary Table 5 **Discriminatory taxa of Anhoni and Tattapani identified using Random Forest Analysis**.

## References

[B1] Afshar S. Johnson E. de Vries S. Schroder I. (2001). Properties of a thermostable nitrate reductase from the hyperthermophilic archaeon *Pyrobaculum aerophilum*. J. Bacteriol. 183, 5491–5495. 10.1128/JB.183.19.5491-5495.2001 11544209 PMC95438

[B2] Altschul S. F. Gish W. Miller W. Myers E. W. Lipman D. J. (1990). Basic local alignment search tool. J. Mol. Biol. 215, 403–410. 10.1016/S0022-2836(05)80360-2 2231712

[B3] Asnicar F. Weingart G. Tickle T. L. Huttenhower C. Segata N. (2015). Compact graphical representation of phylogenetic data and metadata with GraPhlAn. PeerJ 3:e1029. 10.7717/peerj.1029 26157614 PMC4476132

[B4] Bao Y.-J. Xu Z. Li Y. Yao Z. Sun J. Song H (2016). High-throughput metagenomic analysis of petroleum-contaminated soil microbiome reveals the versatility in xenobiotic aromatics metabolism. J. Environ. Sci. 10.1016/j.jes.2016.08.022 Available online at: http://www.sciencedirect.com/science/article/pii/S1001074216306155 28571861

[B5] Bhatia S. Batra N. Pathak A. Green S. J. Joshi A. Chauhan A. (2015). Metagenomic evaluation of bacterial and archaeal diversity in the geothermal hot springs of manikaran, India. Genome Announc. 3:e01544-14. 10.1128/genomeA.01544-14 25700403 PMC4335328

[B6] Bisht S. S. Das N. N. Tripathy N. K. (2011). Indian hot-water springs: a bird's eye view. J. Ener. Environ. Carbon Credits 1, 1–15.

[B7] Bowen H. J. M. (1979). Environmental Chemistry of the Elements. London; New York, NY: Academic Press.

[B8] Bowen De Leon K. Gerlach R. Peyton B. M. Fields M. W. (2013). Archaeal and bacterial communities in three alkaline hot springs in Heart Lake Geyser Basin, Yellowstone National Park. Front. Microbiol. 4:330. 10.3389/fmicb.2013.00330 24282404 PMC3824361

[B9] Caporaso J. G. Kuczynski J. Stombaugh J. Bittinger K. Bushman F. D. Costello E. K.. (2010). QIIME allows analysis of high-throughput community sequencing data. Nat. Methods 7, 335–336. 10.1038/nmeth.f.303 20383131 PMC3156573

[B10] Chan C. S. Chan K. G. Tay Y. L. Chua Y. H. Goh K. M. (2015). Diversity of thermophiles in a Malaysian hot spring determined using 16S rRNA and shotgun metagenome sequencing. Front. Microbiol. 6:177. 10.3389/fmicb.2015.00177 25798135 PMC4350410

[B11] Chandrasekharam D. (2005). Geothermal energy resources of India: past and the present, in Proceedings World Geothermal Congress (Antalya).

[B12] Chaudhary N. Sharma A. K. Agarwal P. Gupta A. Sharma V. K. (2015). 16S classifier: a tool for fast and accurate taxonomic classification of 16S rRNA hypervariable regions in metagenomic datasets. PLoS ONE 10:e0116106. 10.1371/journal.pone.0116106 25646627 PMC4315456

[B13] Colman D. R. Jay Z. J. Inskeep W. P. Jennings R. Maas K. R. Rusch D. B.. (2016). Novel, deep-branching heterotrophic bacterial populations recovered from thermal spring metagenomes. Front. Microbiol. 7:304. 10.3389/fmicb.2016.00304 27014227 PMC4791363

[B14] Coman C. Druga B. Hegedus A. Sicora C. Dragos N. (2013). Archaeal and bacterial diversity in two hot spring microbial mats from a geothermal region in Romania. Extremophiles 17, 523–534. 10.1007/s00792-013-0537-5 23568449

[B15] Deckert G. Warren P. V. Gaasterland T. Young W. G. Lenox A. L. Graham D. E.. (1998). The complete genome of the hyperthermophilic bacterium *Aquifex aeolicus*. Nature 392, 353–358. 10.1038/32831 9537320

[B16] DeSantis T. Z. Hugenholtz P. Larsen N. Rojas M. Brodie E. L. Keller K.. (2006). Greengenes, a chimera-checked 16S rRNA gene database and workbench compatible with ARB. Appl. Environ. Microbiol. 72, 5069–5072. 10.1128/AEM.03006-05 16820507 PMC1489311

[B17] Dhakan D. B. Saxena R. Chaudhary N. Sharma V. K. (2016). Draft genome sequence of *Tepidimonas taiwanensis* strain MB2, a chemolithotrophic thermophile isolated from a hot spring in Central India. Genome Announc. 4:e01723-15. 10.1128/genomeA.01723-15 26893423 PMC4759070

[B18] Dhillon A. Teske A. Dillon J. Stahl D. A. Sogin M. L. (2003). Molecular characterization of sulfate-reducing bacteria in the Guaymas Basin. Appl. Environ. Microbiol. 69, 2765–2772. 10.1128/AEM.69.5.2765-2772.2003 12732547 PMC154542

[B19] Duchscherer W. (1983). Geochemical hydrocarbon exploration—a new/old exploration tool. J. Geochem. Explor. 19, 335–336. 10.1016/0375-6742(83)90025-0

[B20] Eloe-Fadrosh E. A. Paez-Espino D. Jarett J. Dunfield P. F. Hedlund B. P. Dekas A. E.. (2016). Global metagenomic survey reveals a new bacterial candidate phylum in geothermal springs. Nat. Commun. 7:10476. 10.1038/ncomms10476 26814032 PMC4737851

[B21] Farooqui M. Hou H. Li G. Machin N. Neville T. Pal A. (2009). Evaluating volcanic reservoirs. Oilfield Rev. 21, 36–47.

[B22] Ferrandi E. E. Sayer C. Isupov M. N. Annovazzi C. Marchesi C. Iacobone G.. (2015). Discovery and characterization of thermophilic limonene-1,2-epoxide hydrolases from hot spring metagenomic libraries. FEBS J. 282, 2879–2894. 10.1111/febs.13328 26032250

[B23] Gaur N. Flora G. Yadav M. Tiwari A. (2014). A review with recent advancements on bioremediation-based abolition of heavy metals. Environ. Sci. 16, 180–193. 10.1039/c3em00491k 24362580

[B24] Ghelani A. Patel R. Mangrola A. Dudhagara P. (2015). Cultivation-independent comprehensive survey of bacterial diversity in Tulsi Shyam Hot Springs, India. Genom Data 4, 54–56. 10.1016/j.gdata.2015.03.003 26484176 PMC4536058

[B25] Google Maps (2016). Anhoni, Madhya Pradesh to Tattapani, Chhattisgarh. Available online at: https://www.google.co.in/maps/dir/Tattapani,+Chhattisgarh/Anhoni,+Madhya+Pradesh+480559/@23.4008419,79.7856623,6z/data=!4m13!4m12!1m5!1m1!1s0x398be11dce5307e3:0x583e563bac1615ab!2m2!1d83.6840385!2d23.6985978!1m5!1m1!1s0x397e35860faf2e99:0x8f442263b36683f8!2m2!1d78.6058242!2d22.5879396

[B26] Grigoriev I. V. Nordberg H. Shabalov I. Aerts A. Cantor M. Goodstein D.. (2012). The genome portal of the department of energy joint genome institute. Nucleic Acids Res. 40, D26–D32. 10.1093/nar/gkr947 22110030 PMC3245080

[B27] Gudbergsdottir S. R. Menzel P. Krogh A. Young M. Peng X. (2016). Novel viral genomes identified from six metagenomes reveal wide distribution of archaeal viruses and high viral diversity in terrestrial hot springs. Environ. Microbiol. 18, 863–874. 10.1111/1462-2920.13079 26439881

[B28] Gupta A. Kumar S. Prasoodanan V. P. Harish K. Sharma A. K. Sharma V. K. (2016). Reconstruction of bacterial and viral genomes from multiple metagenomes. Front. Microbiol. 7:469. 10.3389/fmicb.2016.00469 27148174 PMC4828583

[B29] Gupta A. Sharma V. K. (2015). Using the taxon-specific genes for the taxonomic classification of bacterial genomes. BMC Genomics 16:1. 10.1186/s12864-015-1542-0 25990029 PMC4438512

[B30] Hannigan R. E. Basu A. R. (1998). Late diagenetic trace element remobilization in organic-rich black shales of the Taconic foreland basin of Quebec, Ontario and New York, in Shales and Mudstones II, eds Zimmerle W. Sethi P. S. (Zurich: Schweizerbart'sche), 209–234.

[B31] Hannigan R. E. Basu A. R. Teichmann F. (2001). Mantle reservoir geochemistry from statistical analysis of ICP-MS trace element data of equatorial mid-Atlantic MORB glasses. Chem. Geol. 175, 397–428. 10.1016/S0009-2541(00)00335-1

[B32] Head I. M. Jones D. M. Röling W. F. (2006). Marine microorganisms make a meal of oil. Nat. Rev. Microbiol. 4, 173–182. 10.1038/nrmicro1348 16489346

[B33] Heulin T. Barakat M. Christen R. Lesourd M. Sutra L. De Luca G.. (2003). *Ramlibacter tataouinensis* gen. nov., sp. nov., and *Ramlibacter henchirensis* sp. nov., cyst-producing bacteria isolated from subdesert soil in Tunisia. Int. J. Syst. Evol. Microbiol. 53(Pt 2), 589–594. 10.1099/ijs.0.02482-0 12710631

[B34] Jimenez D. J. Andreote F. D. Chaves D. Montana J. S. Osorio-Forero C. Junca H.. (2012). Structural and functional insights from the metagenome of an acidic hot spring microbial planktonic community in the Colombian Andes. PLoS ONE 7:e52069. 10.1371/journal.pone.0052069 23251687 PMC3522619

[B35] Kanehisa M. Goto S. (2000). KEGG: kyoto encyclopedia of genes and genomes. Nucleic Acids Res. 28, 27–30. 10.1093/nar/28.1.27 10592173 PMC102409

[B36] Kanoksilapatham W. Keawram P. Gonzalez J. M. Robb F. T. (2015). Isolation, characterization, and survival strategies of *Thermotoga* sp. strain PD524, a hyperthermophile from a hot spring in Northern Thailand. Extremophiles 19, 853–861. 10.1007/s00792-015-0761-2 26101016

[B37] Kent W. J. (2002). BLAT—the BLAST-like alignment tool. Genome Res. 12, 656–664. 10.1101/gr.229202 11932250 PMC187518

[B38] Khan S. D. Jacobson S. (2008). Remote sensing and geochemistry for detecting hydrocarbon microseepages. Geol. Soc. Am. Bull. 120, 96–105. 10.1130/0016-7606(2008)120[96:RSAGFD]2.0.CO;2

[B39] Kim J.-S. Kluskens L. D. de Vos W. M. Huber R. van der Oost J. (2004). Crystal structure of fervidolysin from *Fervidobacterium pennivorans*, a keratinolytic enzyme related to subtilisin. J. Mol. Biol. 335, 787–797. 10.1016/j.jmb.2003.11.006 14687574

[B40] Kimes N. E. Callaghan A. V. Aktas D. F. Smith W. L. Sunner J. Golding B. T.. (2013). Metagenomic analysis and metabolite profiling of deep–sea sediments from the Gulf of Mexico following the Deepwater Horizon oil spill. Front. Microbiol. 4:50. 10.3389/fmicb.2013.00050 23508965 PMC3598227

[B41] Lalucat J. Bennasar A. Bosch R. Garcia-Valdes E. Palleroni N. J. (2006). Biology of *Pseudomonas stutzeri*. Microbiol. Mol. Biol. Rev. 70, 510–547. 10.1128/MMBR.00047-05 16760312 PMC1489536

[B42] Langmead B. Salzberg S. L. (2012). Fast gapped-read alignment with Bowtie 2. Nat. Methods 9, 357–359. 10.1038/nmeth.1923 22388286 PMC3322381

[B43] Li S. J. Hua Z. S. Huang L. N. Li J. Shi S. H. Chen L. X.. (2014). Microbial communities evolve faster in extreme environments. Sci. Rep. 4:6205. 10.1038/srep06205 25158668 PMC4145313

[B44] Liaw A. Wiener M. (2002). Classification and regression by randomForest. R News 2, 18–22. 23795347

[B45] Littlechild J. A. (2015). Enzymes from extreme environments and their industrial applications. Front. Bioeng. Biotechnol. 3:161. 10.3389/fbioe.2015.00161 26528475 PMC4602302

[B46] Lopez-Lopez O. Cerdan M. E. Gonzalez-Siso M. I. (2013). Hot spring metagenomics. Life 3, 308–320. 10.3390/life3020308 25369743 PMC4187134

[B47] Magoc T. Salzberg S. L. (2011). FLASH: fast length adjustment of short reads to improve genome assemblies. Bioinformatics 27, 2957–2963. 10.1093/bioinformatics/btr507 21903629 PMC3198573

[B48] Mangrola A. Dudhagara P. Koringa P. Joshi C. G. Parmar M. Patel R. (2015). Deciphering the microbiota of Tuwa hot spring, India using shotgun metagenomic sequencing approach. Genom Data 4, 153–155. 10.1016/j.gdata.2015.04.014 26484204 PMC4535658

[B49] Mardanov A. V. Gumerov V. M. Beletsky A. V. Perevalova A. A. Karpov G. A. Bonch-Osmolovskaya E. A.. (2011). Un archaea dominate in the thermal groundwater of Uzon Caldera, Kamchatka. Extremophiles 15, 365–372. 10.1007/s00792-011-0368-1 21512891

[B50] Martin M. (2011). Cutadapt removes adapter sequences from high-throughput sequencing reads. EMBnet. J. 17, 10–12. 10.14806/ej.17.1.200

[B51] Mehetre G. T. Paranjpe A. S. Dastager S. G. Dharne M. S. (2016). Complete metagenome sequencing based bacterial diversity and functional insights from basaltic hot spring of Unkeshwar, Maharashtra, India. Genom Data 7, 140–143. 10.1016/j.gdata.2015.12.031 26981391 PMC4778638

[B52] Menzel P. Gudbergsdottir S. R. Rike A. G. Lin L. Zhang Q. Contursi P.. (2015). Comparative metagenomics of eight geographically remote terrestrial hot springs. Microb. Ecol. 70, 411–424. 10.1007/s00248-015-0576-9 25712554

[B53] Mirete S. Morgante V. Gonzalez-Pastor J. E. (2016). Functional metagenomics of extreme environments. Curr. Opin. Biotechnol. 38, 143–149. 10.1016/j.copbio.2016.01.017 26901403

[B54] Mongenot T. Tribovillard N.-P. Desprairies A. Lallier-Vergès E. Laggoun-Defarge F. (1996). Trace elements as palaeoenvironmental markers in strongly mature hydrocarbon source rocks: the Cretaceous La Luna Formation of Venezuela. Sediment. Geol. 103, 23–37. 10.1016/0037-0738(95)00078-X

[B55] Moriya Y. Itoh M. Okuda S. Yoshizawa A. C. Kanehisa M. (2007). KAAS: an automatic genome annotation and pathway reconstruction server. Nucleic Acids Res. 35, W182–W185. 10.1093/nar/gkm321 17526522 PMC1933193

[B56] Namiki T. Hachiya T. Tanaka H. Sakakibara Y. (2012). MetaVelvet: an extension of Velvet assembler to *de novo* metagenome assembly from short sequence reads. Nucleic Acids Res. 40, e155. 10.1093/nar/gks678 22821567 PMC3488206

[B57] Oksanen J. Blanchet F. G. Kindt R. Legendre P. Minchin P. R. O'Hara R. (2013). Package ‘Vegan.’ Community Ecology Package, Version 2.

[B58] Oliveira A. P. Patil K. R. Nielsen J. (2008). Architecture of transcriptional regulatory circuits is knitted over the topology of bio-molecular interaction networks. BMC Syst. Biol. 2:17. 10.1186/1752-0509-2-17 18261202 PMC2268660

[B59] Orphan V. J. Taylor L. T. Hafenbradl D. Delong E. F. (2000). Culture-dependent and culture-independent characterization of microbial assemblages associated with high-temperature petroleum reservoirs. Appl. Environ. Microbiol. 66, 700–711. 10.1128/AEM.66.2.700-711.2000 10653739 PMC91884

[B60] Pal P. Bhimasankaram V. (1976). Tectonics of the Narmada-Son-Brahmaputra lineament. Misc. Publ Geol. Surv. Ind 34, 133–140.

[B61] Pandey O. Negi J. (1995). Geothermal fields of India: a latest update, in Proceedings World Geothermal Congress (Florence), 163–171.

[B62] Patel R. K. Jain M. (2012). NGS QC Toolkit: a toolkit for quality control of next generation sequencing data. PLoS ONE 7:e30619. 10.1371/journal.pone.0030619 22312429 PMC3270013

[B63] Poli A. Nicolaus B. Chan K. G. Kahar U. M. Chan C. S. Goh K. M. (2015). Genome Sequence of *Anoxybacillus thermarum* AF/04^T^, isolated from the euganean hot springs in Abano Terme, Italy. Genome Announc. 3:e00490-15. 10.1128/genomeA.00490-15 25999577 PMC4440957

[B64] Powell S. Forslund K. Szklarczyk D. Trachana K. Roth A. Huerta-Cepas J.. (2014). eggNOG v4.0: nested orthology inference across 3686 organisms. Nucleic Acids Res. 42, D231–D239. 10.1093/nar/gkt1253 24297252 PMC3964997

[B65] Price L. C. (1986). A critical overview and proposed working model of surface geochemical exploration, in Unconventional Methods in Exploration for Petroleum and Natural Gas, Symposium IV. Dallas, TX: Southern Methodist University Press.

[B66] Sarolkar P. (2010). Exploration strategy for hot springs associated with gondwana coalfields in India. Proc. World Geother. Congr. 74, 68.

[B67] Sarolkar P. (2015). Strategy for development of geothermal resources in India, in Proceedings World Geothermal Congress (Melbourne, VIC).

[B68] Sarolkar P. Das A. (2006). Reservoir studies at Tatapani geothermal field, Surguja district, India, in Proceedings of 31st Workshop on Geothermal Reservoir Engineering (Stanford, CA, Stanford University, SGP-TR-179).

[B69] Saxena R. Chaudhary N. Dhakan D. B. Sharma V. K. (2015). Draft genome sequence of *Gulbenkiania mobilis* strain MB1, a sulfur-metabolizing thermophile isolated from a hot spring in Central India. Genome Announc. 3:e01295-15. 10.1128/genomeA.01295-15 26586874 PMC4653776

[B70] Shanker R. (1986). Scope of utilisation of geothermal energy for area development in backward, Hilly and Tribal regions of India. Indian Miner. 40, 49–61.

[B71] Sharma A. Hira P. Shakarad M. Lal R. (2014). Draft genome sequence of *Cellulosimicrobium* sp. Strain MM, isolated from arsenic-rich microbial mats of a himalayan hot spring. Genome Announc. 2:e01020-14. 10.1128/genomeA.01020-14 25301656 PMC4192388

[B72] Singleton D. R. Ramirez L. G. Aitken M. D. (2009). Characterization of a polycyclic aromatic hydrocarbon degradation gene cluster in a phenanthrene-degrading Acidovorax strain. Appl. Environ. Microbiol. 75, 2613–2620. 10.1128/AEM.01955-08 19270134 PMC2681696

[B73] Soergel D. A. Dey N. Knight R. Brenner S. E. (2012). Selection of primers for optimal taxonomic classification of environmental 16S rRNA gene sequences. ISME J. 6, 1440–1444. 10.1038/ismej.2011.208 22237546 PMC3379642

[B74] Stetter K. O. (1996). Hyperthermophilic procaryotes. FEMS Microbiol. Rev. 18, 149–158. 10.1111/j.1574-6976.1996.tb00233.x

[B75] Stetter K. O. (1999). Extremophiles and their adaptation to hot environments. FEBS Lett. 452, 22–25. 10.1016/S0014-5793(99)00663-8 10376671

[B76] Tedesco S. A. (2012). Surface Geochemistry in Petroleum Exploration. New York, NY: Springer Science & Business Media.

[B77] Tekere M. Lötter A. Olivier J. Jonker N. Venter S. (2011). Metagenomic analysis of bacterial diversity of Siloam hot water spring, Limpopo, South Africa. Afr. J. Biotechnol. 10, 18005–18012. 10.5897/AJB11.899

[B78] Tindall B. J. Sikorski J. Lucas S. Goltsman E. Copeland A. Del Rio T. G.. (2010). Complete genome sequence of *Meiothermus ruber* type strain (21 T). Stand. Genomic Sci. 3, 26. 10.4056/sigs.1032748 21304689 PMC3035268

[B79] Vaidya D. Shah M. Sircar A. Sahajpal S. Dhale S. (2015). Geothermal energy: exploration efforts in India. Int. J. Latest Res. Sci. Technol. 4, 61–69.

[B80] Wang Y. Qian P. Y. (2009). Conservative fragments in bacterial 16S rRNA genes and primer design for 16S ribosomal DNA amplicons in metagenomic studies. PLoS ONE 4:e7401. 10.1371/journal.pone.0007401 19816594 PMC2754607

[B81] Wang Y. Wang X. Tang R. Yu S. Zheng B. Feng Y. (2010). A novel thermostable cellulase from *Fervidobacterium nodosum*. J. Mol. Catal. B Enzym. 66, 294–301. 10.1016/j.molcatb.2010.06.006

[B82] Ward N. Larsen O. Sakwa J. Bruseth L. Khouri H. Durkin A. S.. (2004). Genomic insights into methanotrophy: the complete genome sequence of *Methylococcus capsulatus* (Bath). PLoS Biol. 2:e303. 10.1371/journal.pbio.0020303 15383840 PMC517821

[B83] Xie W. Wang F. Guo L. Chen Z. Sievert S. M. Meng J.. (2011). Comparative metagenomics of microbial communities inhabiting deep-sea hydrothermal vent chimneys with contrasting chemistries. ISME J. 5, 414–426. 10.1038/ismej.2010.144 20927138 PMC3105715

[B84] Zhu W. Lomsadze A. Borodovsky M. (2010). Ab initio gene identification in metagenomic sequences. Nucleic Acids Res. 38:e132. 10.1093/nar/gkq275 20403810 PMC2896542

