# Supplementary figures and images for: Metagenomic Analysis of Hot Springs in Central India Reveals Hydrocarbon Degrading Thermophiles and Pathways Essential for Survival in Extreme Environments
Source: Front Microbiol. 2017 Jan 5;7:2123. doi: 10.3389/fmicb.2016.02123 (PMC5214690; doi:10.3389/fmicb.2016.02123)

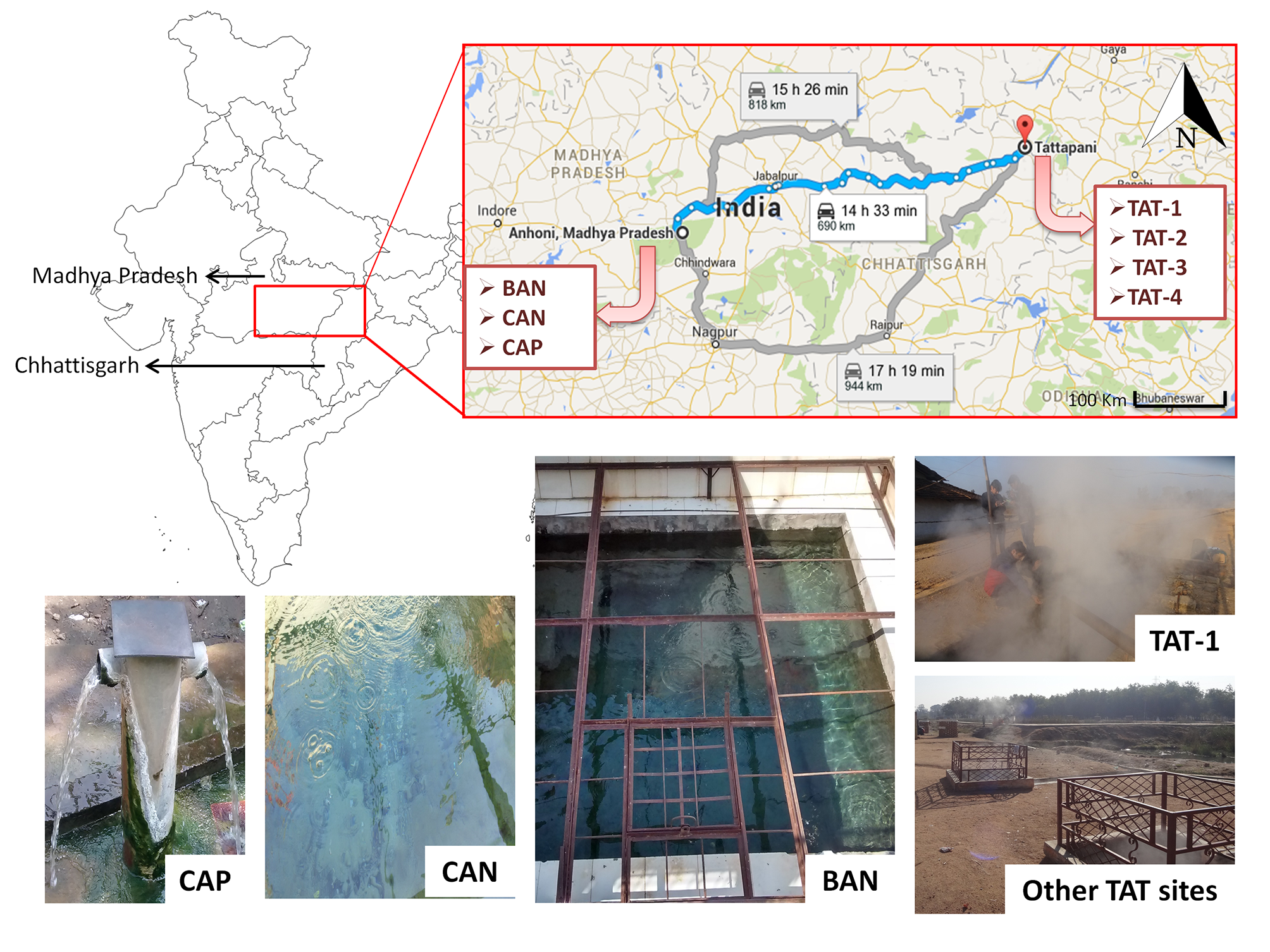

Supplement: Supplementary Figure 1 — Geographical location and photographs of sampling sites of Anhoni and Tattapani hot springs. The given map was retrieved from Google Maps (2016). [file Image1.TIF]

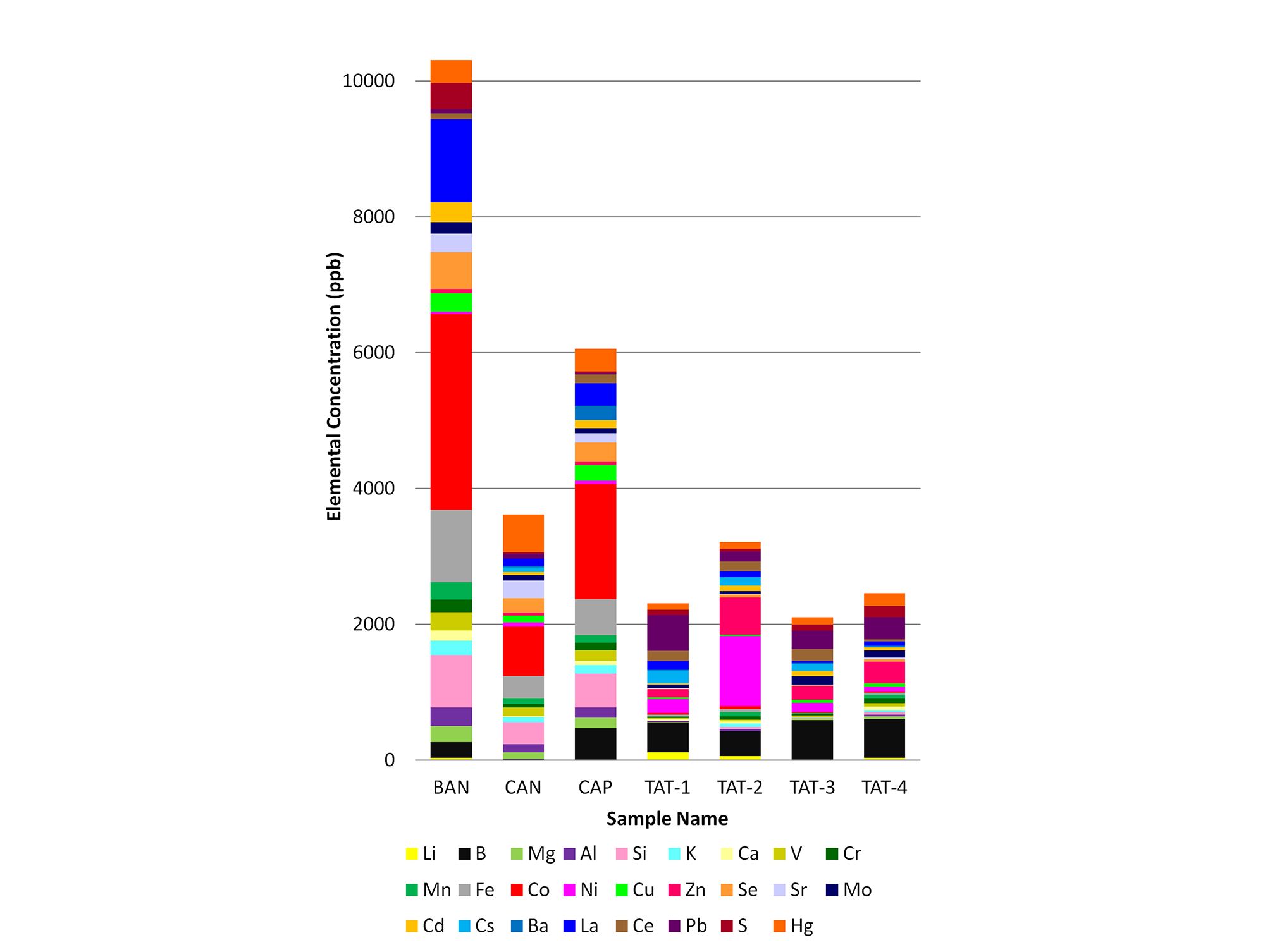

Supplement: Supplementary Figure 2 — Graphical representation of elemental composition at the seven sample sites. [file Image2.TIF]

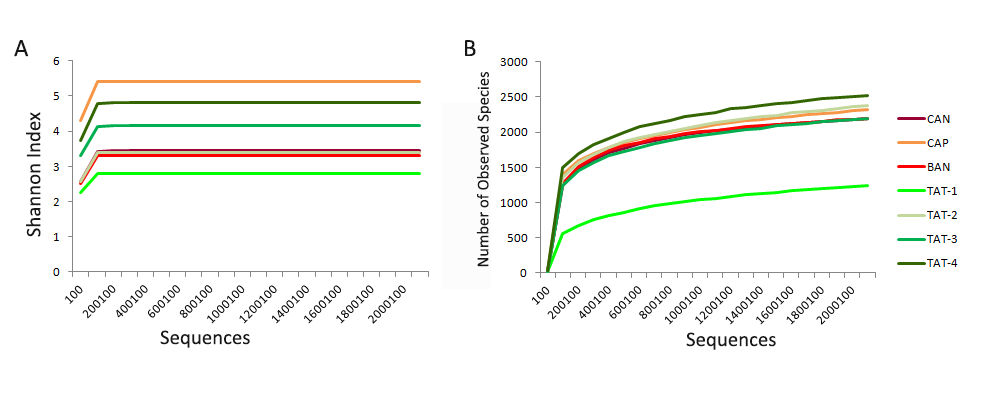

Supplement: Supplementary Figure 3 — Alpha diversity in the Hot spring samples. (A) Shannon index and (B) Observed Species were calculated by rarefying from 100 to 2.2 million sequences at a step size of 0.1 million. This analysis was carried out using amplicon reads. [file Image3.TIF]

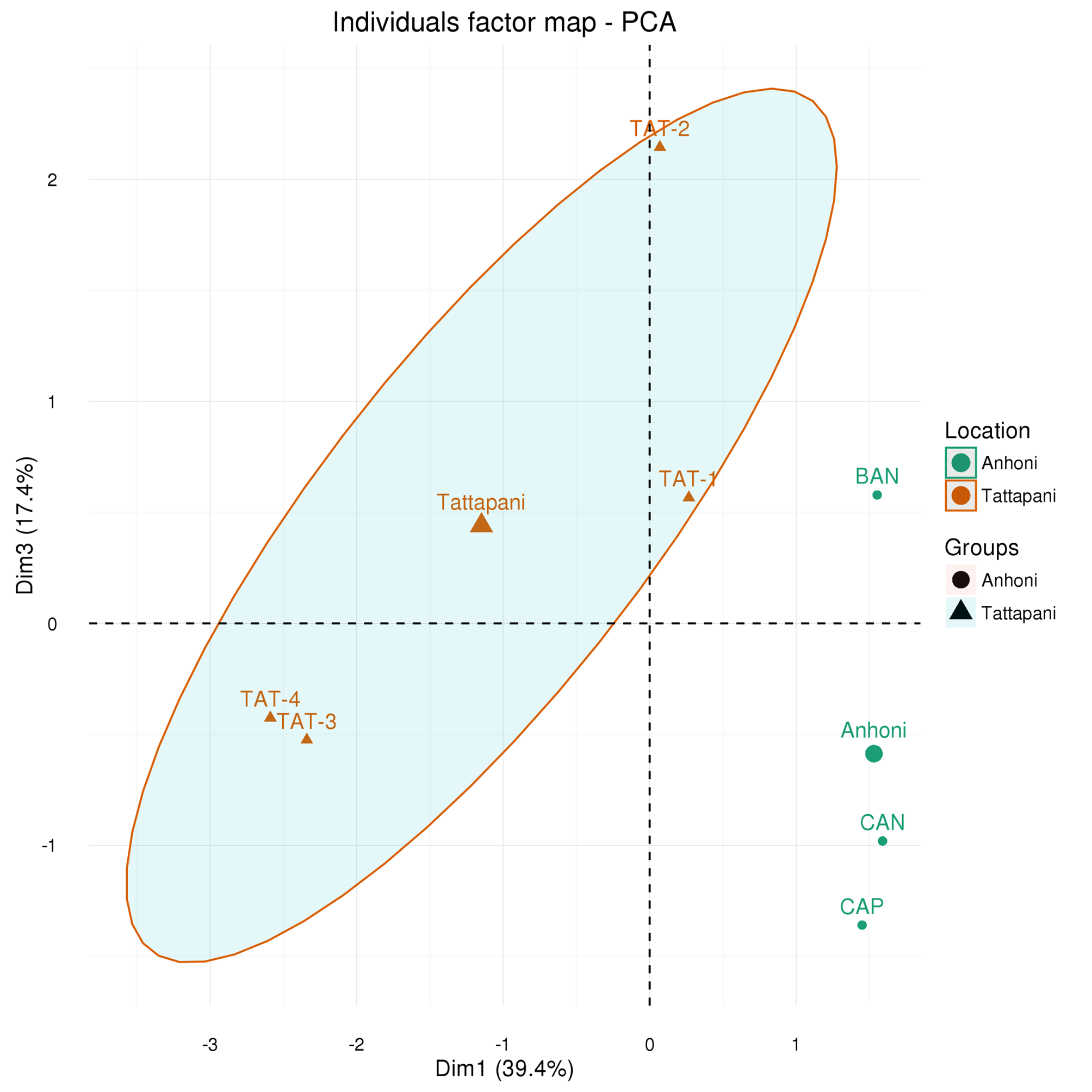

Supplement: Supplementary Figure 4 — PCA plot prepared using Hellinger distances based on eggNOG proportions. [file Image4.TIFF]

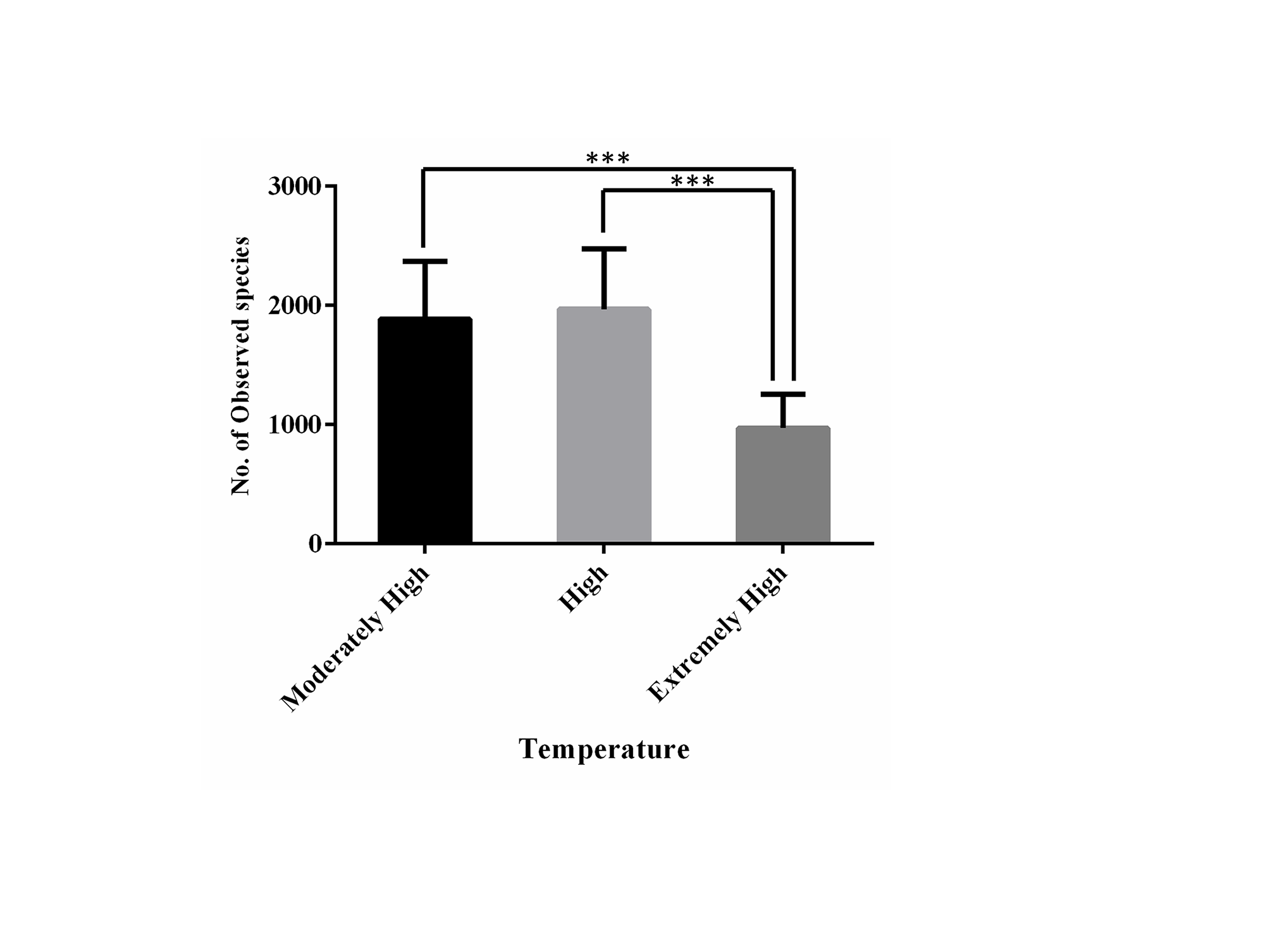

Supplement: Supplementary Figure 5 — Multiple comparisons of number of observed species with moderately high, high and extremely high-temperature locations. Multiple comparisons using Tukey's test were performed between samples grouped on the basis of temperature (moderately high, high, and extremely high). The mean ± SD are plotted with significant variations shown as ***p ≤ 0.001. [file Image5.TIF]

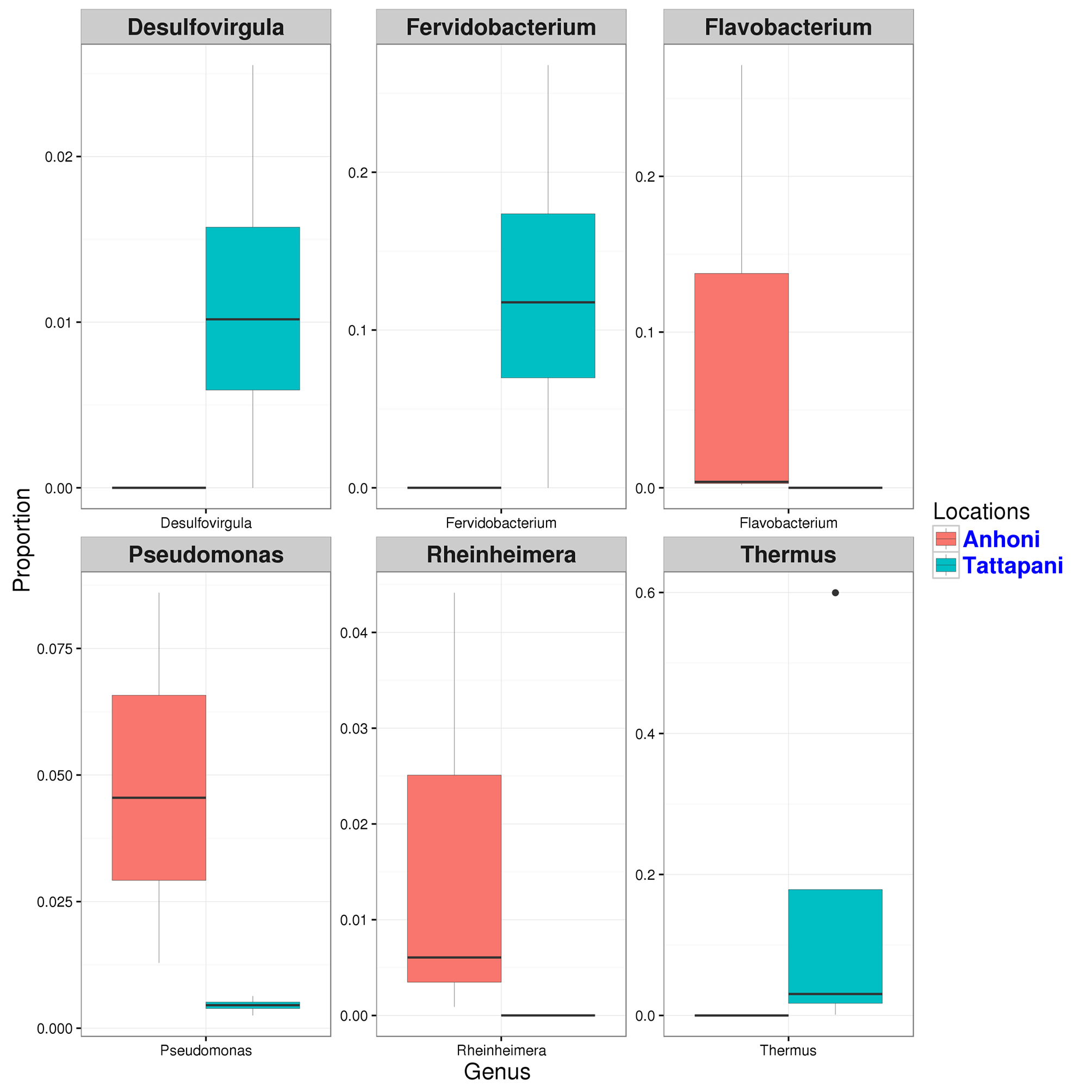

Supplement: Supplementary Figure 6 — Significantly discriminatory genera (p ≤ 0.05) in Anhoni and Tattapani sites. [file Image6.TIFF]

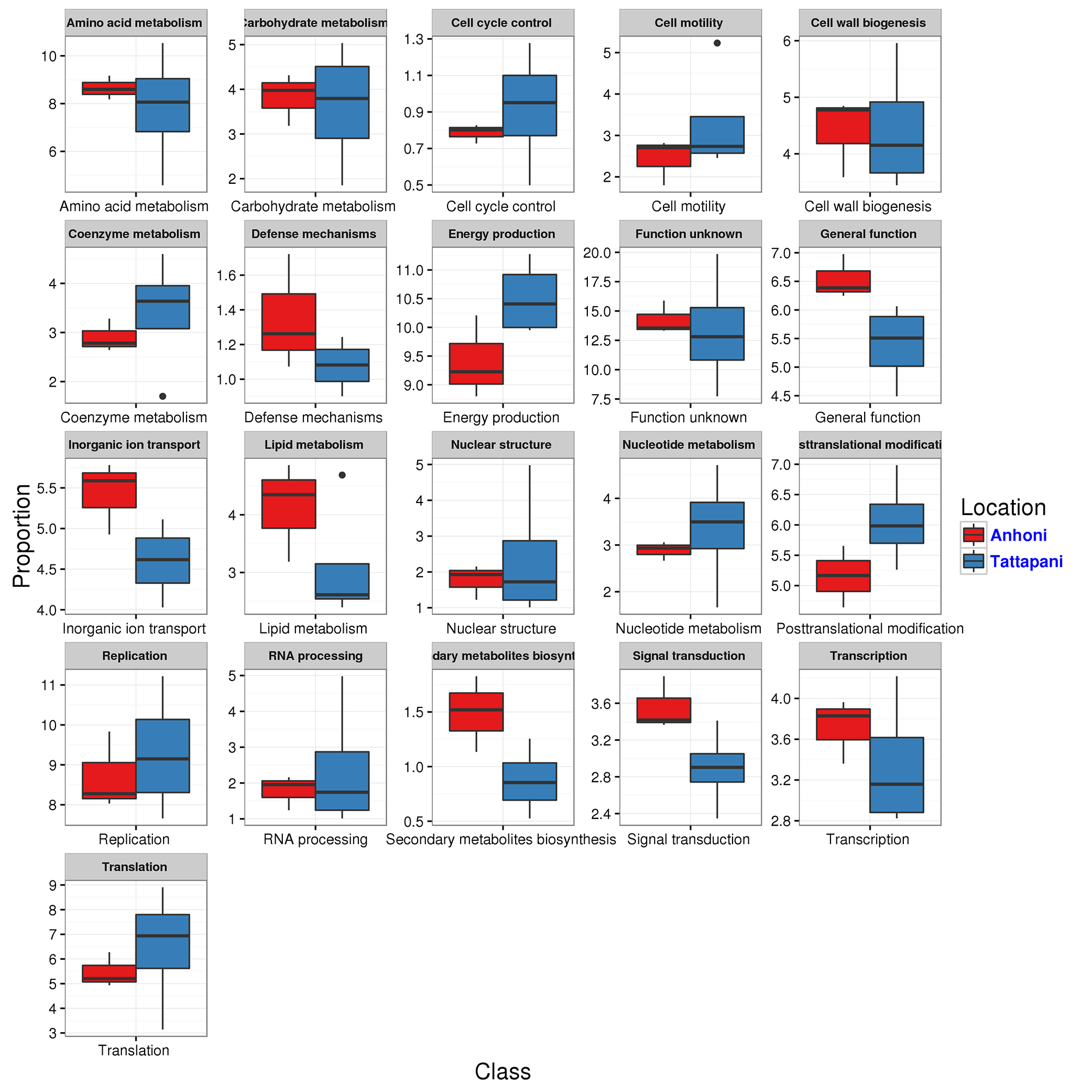

Supplement: Supplementary Figure 7 — eggNOG functional classes and their distributions in the two sites. The box plot shows the abundance of eggNOG functional categories in Anhoni and Tattapani. [file Image7.TIF]

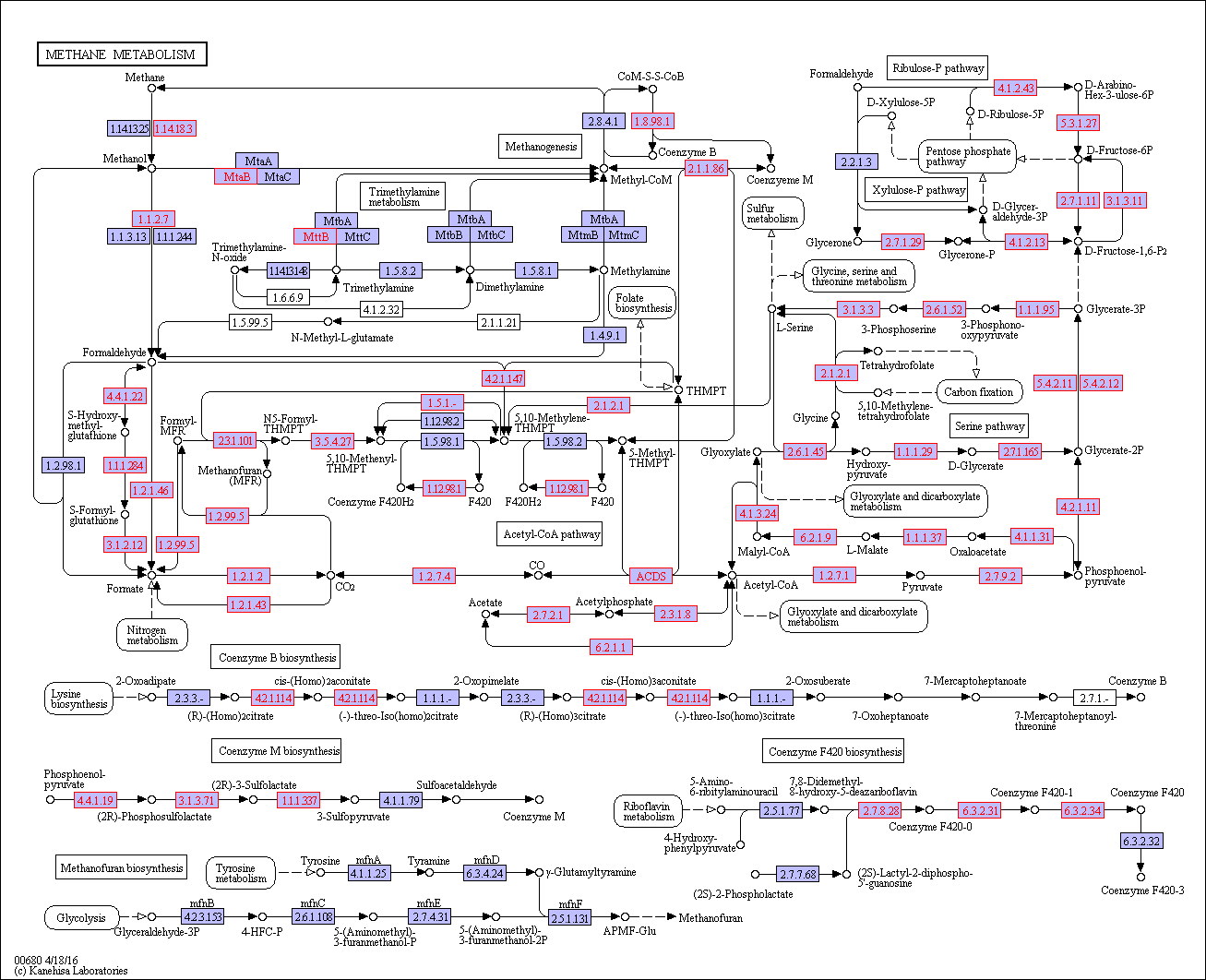

Supplement: Supplementary Figure 8 — A pathway map of methane metabolism showing the observed KOs (highlighted in red) in this dataset. Methane is oxidized to methanol via methane monooxygenase and converted to formaldehyde with the help of methanol dehydrogenase. The KOs which are commonly present in all three sites (CAP, CAN, and BAN) of the Anhoni region are shown. [file Image8.PNG]

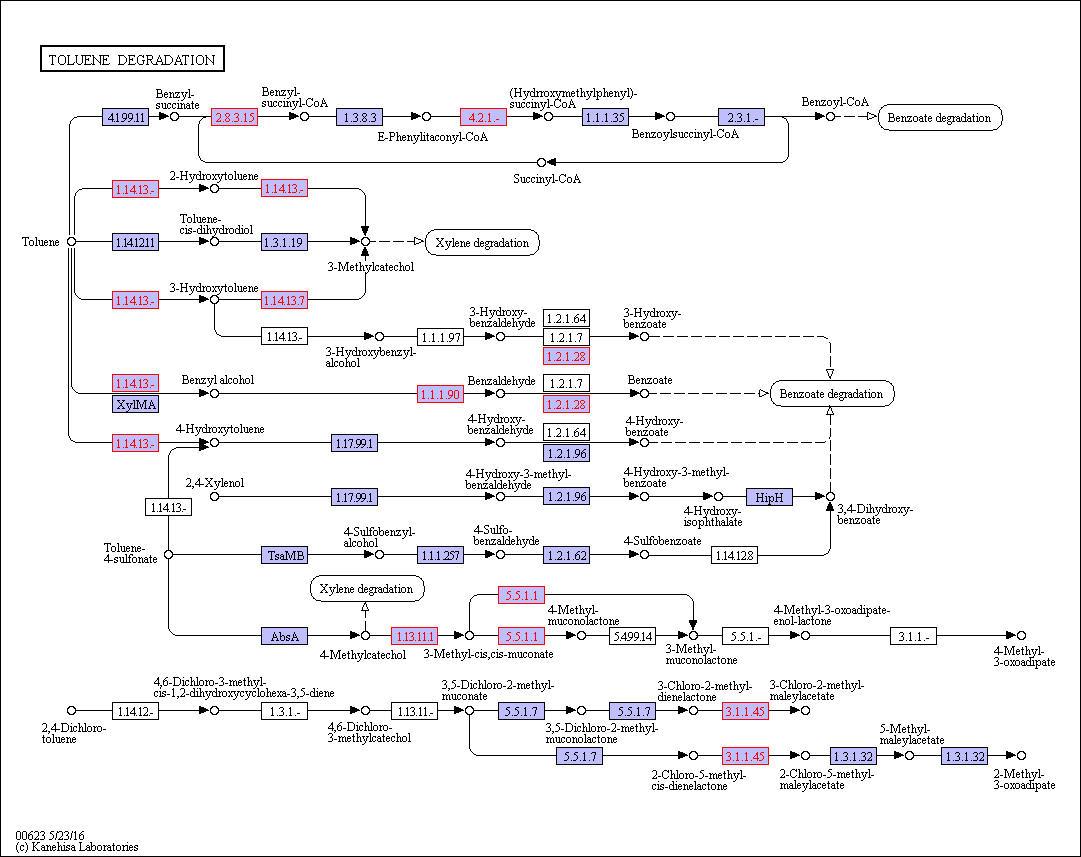

Supplement: Supplementary Figure 9 — A pathway map of toluene degradation showing the observed KOs (highlighted in red) in this dataset. Toluene is degraded to Benzoate and 3-methylcatechol which enters the benzoate degradation and xylene degradation pathways respectively for further downstream processes. The KOs which are commonly present in all three sites (CAP, CAN, and BAN) of the Anhoni region are shown. [file Image9.PNG]

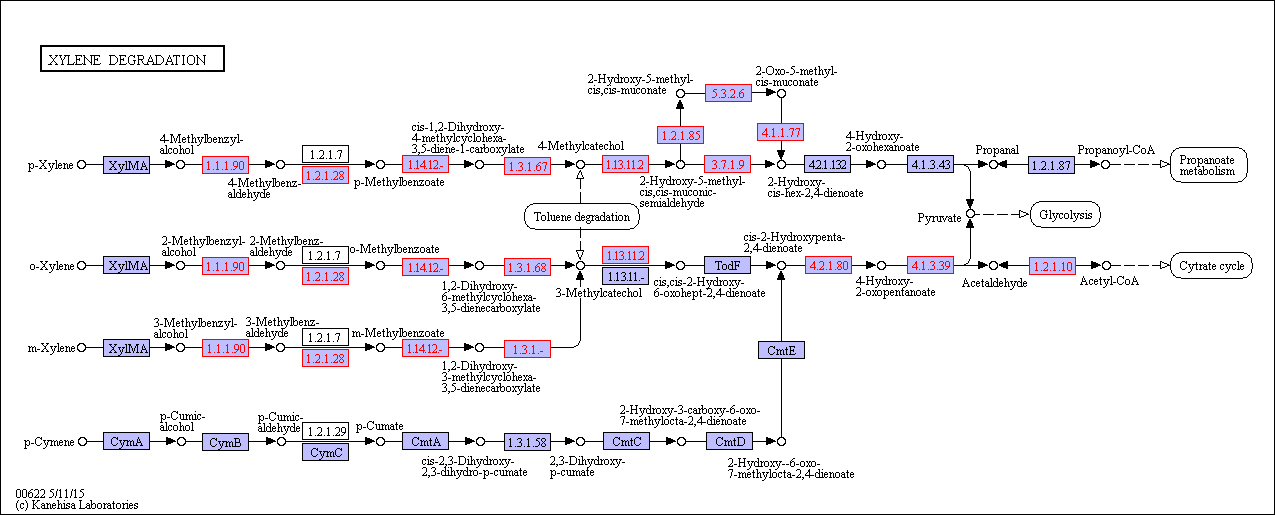

Supplement: Supplementary Figure 10 — A pathway map of xylene degradation showing the KOs (highlighted in red) observed in our dataset. Xylene is degraded and converted to Acetyl-CoA by the community microbes and enters the TCA cycle for energy generation. The KOs which are commonly present in all three sites (CAP, CAN, and BAN) of the Anhoni region are shown. [file Image10.PNG]

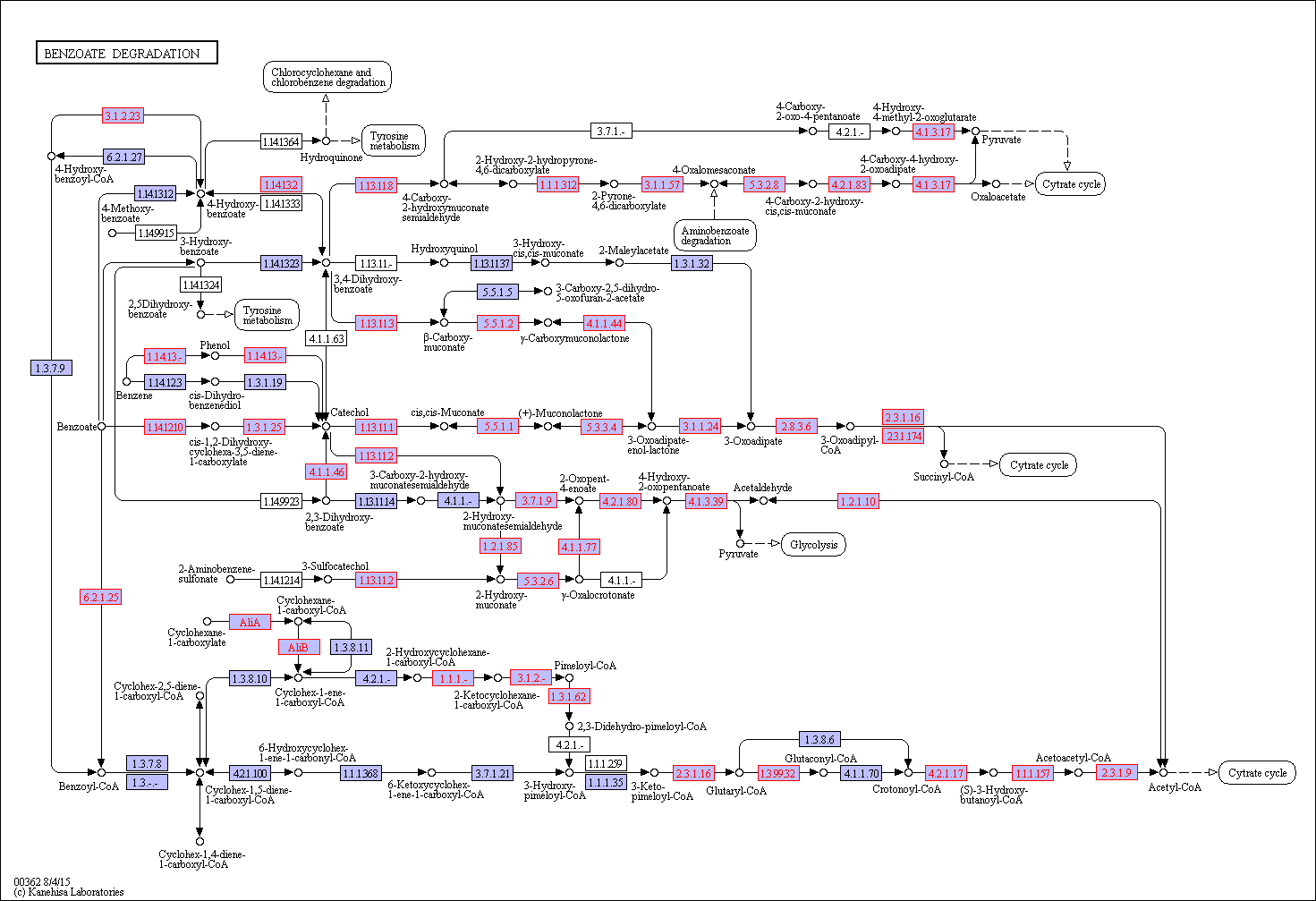

Supplement: Supplementary Figure 11 — A pathway map of benzoate degradation showing the observed KOs (highlighted in red) in this dataset. Benzoate is degraded and converted to intermediates such as Pyruvate and Succinyl-CoA, finally entering into TCA cycle for energy generation. The KOs which are commonly present in all three sites (CAP, CAN, and BAN) of the Anhoni region are shown. [file Image11.PNG]

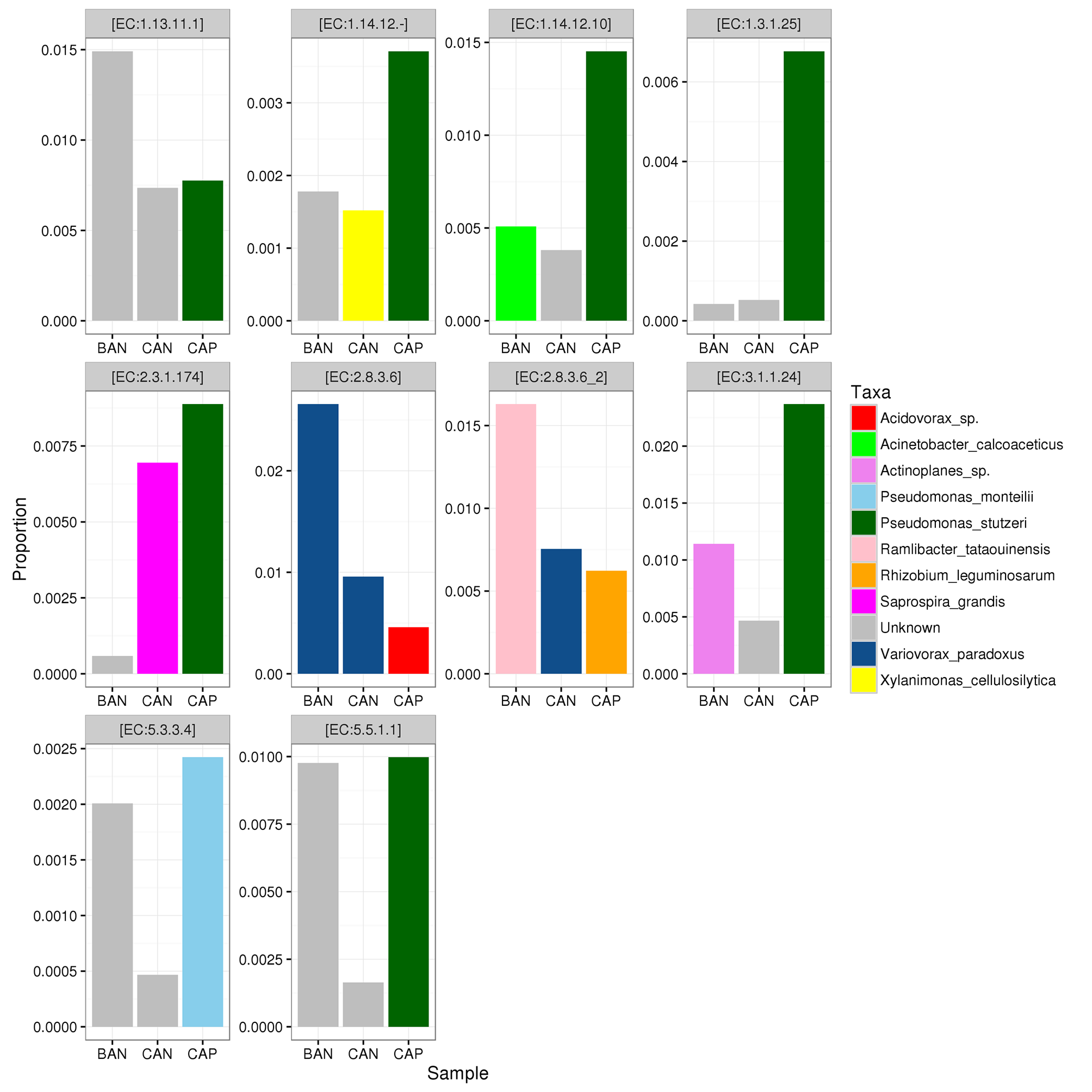

Supplement: Supplementary Figure 12 — Bar charts showing the proportion of key enzymes of benzoate pathway observed in this dataset along with their taxonomic annotations. [file Image12.TIFF]

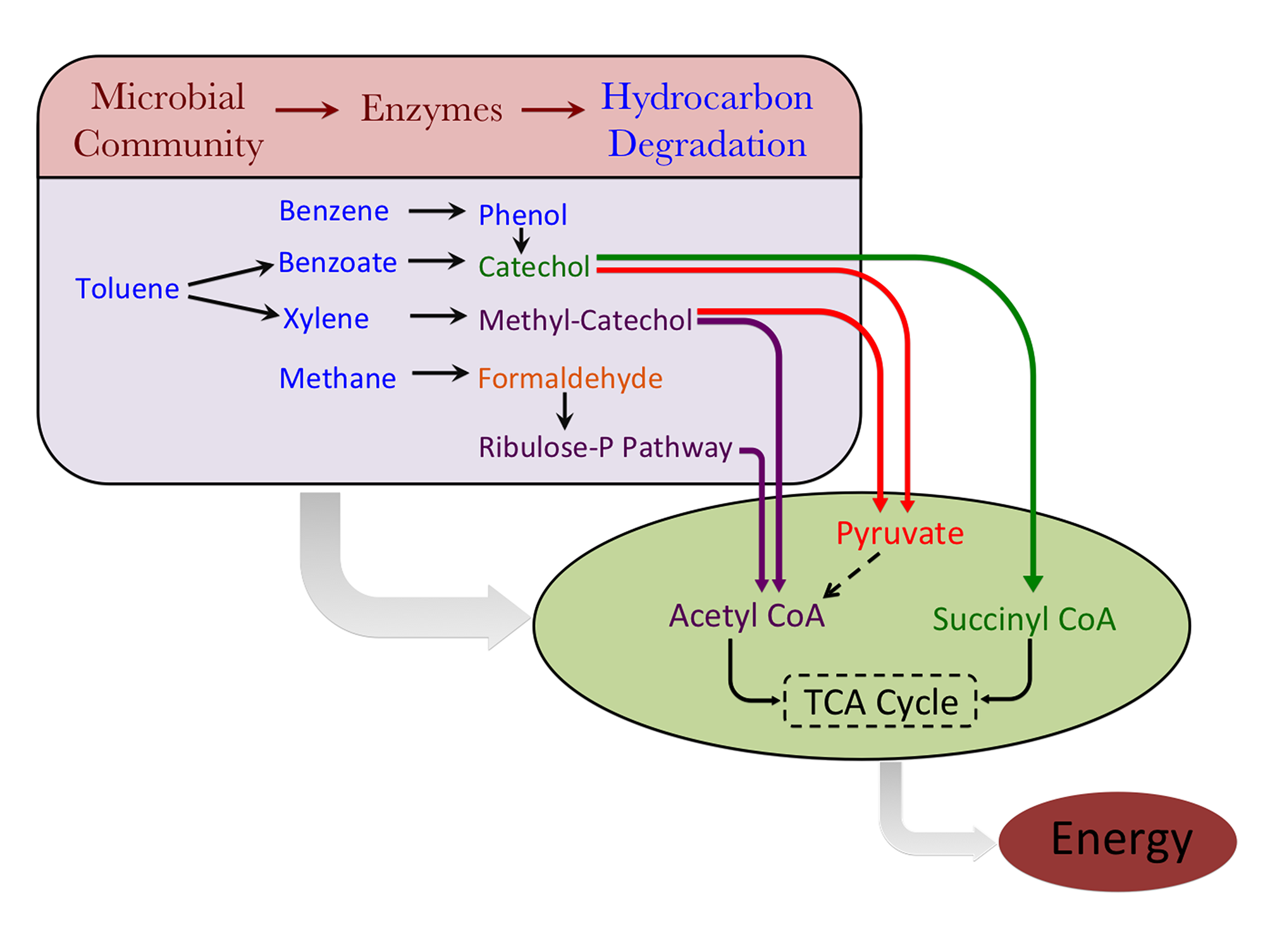

Supplement: Supplementary Figure 13 — Utilization of hydrocarbons by the microbial community for generation of energy. The hydrocarbons are utilized as a substrate by the chemoorganotrophic thermophiles to produce energy. The degradation pathways which were observed to be completely present in Anhoni samples are shown with some of the intermediates and their end-products. [file Image13.TIF]
