# Supplementary material for: Metagenomic Analysis of Hot Springs in Central India Reveals Hydrocarbon Degrading Thermophiles and Pathways Essential for Survival in Extreme Environments
Source: Front Microbiol. 2017 Jan 5;7:2123. doi: 10.3389/fmicb.2016.02123 (PMC5214690; doi:10.3389/fmicb.2016.02123)
Supplement: Supplementary Table 1 — Elemental analysis of the hot spring samples. [file Table1.DOCX]

**Supplementary Table 1. Elemental analysis of the hot spring samples**

| **Element*** | **BAN** | **CAN** | **CAP** | **TAT-1** | **TAT-2** | **TAT-3** | **TAT-4** |
| --- | --- | --- | --- | --- | --- | --- | --- |
| Li | 34.41 | 7.96 | 4.80 | 119.74 | 61.83 | ND | 32.86 |
| B | 232.97 | 17.01 | 468.97 | 425.45 | 365.34 | 591.94 | 573.43 |
| Mg | 233.21 | 94.02 | 153.36 | 16.60 | 2.58 | 24.40 | 37.55 |
| Al | 278.74 | 115.34 | 150.20 | 16.22 | 29.78 | 0.76 | 28.28 |
| Si | 767.65 | 322.40 | 500.77 | 8.64 | 29.98 | 9.43 | 38.06 |
| K | 211.34 | 74.93 | 124.61 | ND | 50.04 | 4.84 | 27.52 |
| Ca | 151.05 | 23.37 | 58.34 | 23.91 | 32.58 | 3.81 | 50.24 |
| V | 267.59 | 120.42 | 156.03 | 9.31 | 22.88 | 26.03 | 49.03 |
| Cr | 189.14 | 53.77 | 110.86 | 18.54 | 53.77 | 23.27 | 75.69 |
| Mn | 255.74 | 83.51 | 112.84 | 7.42 | 58.39 | 9.99 | 49.61 |
| Fe | 1063.70 | 322.39 | 532.30 | 27.47 | 47.83 | 0.43 | 32.10 |
| Co | 2882.70 | 733.48 | 1693.34 | 23.57 | 42.23 | 15.94 | 18.40 |
| Ni | 29.34 | 57.82 | 49.41 | 205.91 | 1029.91 | 132.13 | 67.16 |
| Cu | 276.67 | 102.69 | 228.06 | 22.03 | 22.03 | 47.00 | 50.48 |
| Zn | 59.34 | 45.50 | 42.42 | 121.18 | 547.46 | 203.98 | 320.77 |
| Se | 541.74 | 207.71 | 288.15 | 5.78 | 45.14 | 4.91 | 37.31 |
| Sr | 275.45 | 263.11 | 139.78 | 9.70 | 6.67 | 15.16 | 23.05 |
| Mo | 171.66 | 82.98 | 72.55 | 48.94 | 41.77 | 125.32 | 103.83 |
| Cd | 287.93 | 39.69 | 118.40 | 22.85 | 78.35 | 75.08 | 48.97 |
| Cs | 0.32 | 68.28 | 2.54 | 183.39 | 125.48 | 106.17 | 0.00 |
| Ba | 1.27 | 21.69 | 207.96 | 14.80 | 1.85 | 7.40 | 24.05 |
| La | 1221.05 | 111.01 | 333.02 | 132.14 | 84.57 | 37.00 | 58.14 |
| Ce | 85.10 | 1.85 | 131.35 | 148.00 | 148.00 | 173.62 | 31.31 |
| Pb | 66.26 | 66.26 | 19.79 | 525.35 | 140.57 | 273.76 | 325.58 |
| S | 387.25 | 24.99 | 23.72 | 81.54 | 42.69 | 88.15 | 169.35 |
| Hg | 333.00 | 555.00 | 333.00 | 89.86 | 100.43 | 100.43 | 185.00 |

*: The concentration is measured in parts per billion

ND: Not detectable (<0.5 ppb)
