# Supplementary material for: Metagenomic Analysis of Hot Springs in Central India Reveals Hydrocarbon Degrading Thermophiles and Pathways Essential for Survival in Extreme Environments
Source: Front Microbiol. 2017 Jan 5;7:2123. doi: 10.3389/fmicb.2016.02123 (PMC5214690; doi:10.3389/fmicb.2016.02123)
Supplement: Supplementary Table 2 — Number of 16S rRNA (V3 hypervariable region) amplicon reads obtained per sample. [file Table2.DOCX]

**Supplementary Table 2. Number of 16S rRNA (V3 hyper variable region) amplicon reads obtained per sample**

| **Samples** | **Number of Raw Reads** | **Number of High quality reads** |
| --- | --- | --- |
| **CAP** | 3,874,850 | 3,048,182 |
| **BAN** | 4,382,060 | 3,567,202 |
| **CAN** | 2,896,430 | 2,318,060 |
| **TAT-1** | 4,468,139 | 3,574,921 |
| **TAT-2** | 4,043,404 | 3,041,973 |
| **TAT-3** | 5,151,447 | 3,969,061 |
| **TAT-4** | 3,011,091 | 2,362,487 |
