# Supplementary material for: Metagenomic Analysis of Hot Springs in Central India Reveals Hydrocarbon Degrading Thermophiles and Pathways Essential for Survival in Extreme Environments
Source: Front Microbiol. 2017 Jan 5;7:2123. doi: 10.3389/fmicb.2016.02123 (PMC5214690; doi:10.3389/fmicb.2016.02123)
Supplement: Supplementary Table 3 — Sequencing and assembly statistics of the hot spring samples obtained after de novo assembly. The size of contigs ranged from 1817 to 189,789 bp indicating a taxonomic diversity across different samples. [file Table3.DOCX]

**Supplementary Table 3. Sequencing and assembly statistics of the hot spring samples obtained after *de novo* assembly.** The size of contigs ranged from 1,817 to 189,789 bp indicating a taxonomic diversity across different samples.

| **Samples** | **No. of high quality reads** | **No. of assembled contigs** | **Largest contig** | **N50 contig size** | **Median Coverage depth** | **% GC** |
| --- | --- | --- | --- | --- | --- | --- |
| **CAP** | 19,554,880 | 189,789 | 300,790 | 1,095 | 5.804 | 58.76 |
| **CAN** | 15,400,199 | 35,630 | 308,961 | 1,436 | 5.31 | 56.83 |
| **BAN** | 15,622,317 | 10,034 | 255,192 | 2,243 | 5.95 | 63.36 |
| **TAT-1** | 10,967,018 | 1,817 | 203,188 | 37,514 | 24.59 | 50.5 |
| **TAT-2** | 13,563,485 | 60,087 | 10,394 | 358 | 3.48 | 67.19 |
| **TAT-3** | 15,209,380 | 61,774 | 99,748 | 2,970 | 6.58 | 56.52 |
| **TAT-4** | 8,839,629 | 14,662 | 119,840 | 3,144 | 5.98 | 47.45 |
