# Supplementary material for: Metagenomic Analysis of Hot Springs in Central India Reveals Hydrocarbon Degrading Thermophiles and Pathways Essential for Survival in Extreme Environments
Source: Front Microbiol. 2017 Jan 5;7:2123. doi: 10.3389/fmicb.2016.02123 (PMC5214690; doi:10.3389/fmicb.2016.02123)
Supplement: Supplementary Table 4 — The alpha diversity metrics calculated for 16SrRNA and metagenomics reads are shown for both sites (mean ± SD). [file Table4.DOCX]

**Supplementary Table 4. The alpha diversity metrics calculated for 16SrRNA and metagenomics reads are shown for both sites (mean ± sd).**

| **Diversity** | **Anhoni** | **Tattapani** | **Mann-Whitney U Test** |
| --- | --- | --- | --- |
| **Observed Species (16SrRNA)** | 2239 ± 95.016 | 2086.15 ± 290.220 | 0.745 |
| **Shannon index (16SrRNA)** | 4.056 ± 0.676 | 3.792 ± 0.441 | 0.857 |
| **Pielou’s evenness (16SrRNA)** | 0.362602 ± 0.059272 | 0.341453 ± 0.035272 | 0.857 |
| **Shannon index (Gene Diversity)** | 10.73 ± 0.637 | 9.51 ± 0.22 | **0.0148*** |
| **Pielou’s evenness (Gene Diversity)** | 0.898 ± 0.0193 | 0.892 ± 0.03 | 0.114 |

*: significant (p-value ≤ 0.05)
